# BCAT1 affects mitochondrial metabolism independently of leucine transamination in activated human macrophages

**DOI:** 10.1242/jcs.247957

**Published:** 2020-11-27

**Authors:** Jeong-Hun Ko, Antoni Olona, Adonia E. Papathanassiu, Norzawani Buang, Kwon-Sik Park, Ana S. H. Costa, Claudio Mauro, Christian Frezza, Jacques Behmoaras

**Affiliations:** 1 Centre for Inflammatory Disease, Imperial College London, London W12 0NN, UK; 2 Ergon Pharmaceuticals, LLC, P.O. Box 1001, Silver Spring, MD 20910, USA; 3 Department of Microbiology, Immunology, and Cancer Biology, University of Virginia School of Medicine, Charlottesville, VA 22908, USA; 4 Medical Research Council Cancer Unit, University of Cambridge, Cambridge CB2 0XZ, UK; 5 Institute of Inflammation and Ageing, College of Medical and Dental Sciences, University of Birmingham, Mindelsohn Way, Birmingham B15 2WB, UK

**Keywords:** BCAT1, TCA cycle, Immunometabolism, Macrophages, Mitochondria, Redox biology

## Abstract

In response to environmental stimuli, macrophages change their nutrient consumption and undergo an early metabolic adaptation that progressively shapes their polarization state. During the transient, early phase of pro-inflammatory macrophage activation, an increase in tricarboxylic acid (TCA) cycle activity has been reported, but the relative contribution of branched-chain amino acid (BCAA) leucine remains to be determined. Here, we show that glucose but not glutamine is a major contributor of the increase in TCA cycle metabolites during early macrophage activation in humans. We then show that, although uptake of BCAAs is not altered, their transamination by BCAT1 is increased following 8 h lipopolysaccharide (LPS) stimulation. Of note, leucine is not metabolized to integrate into the TCA cycle in basal or stimulated human macrophages. Surprisingly, the pharmacological inhibition of BCAT1 reduced glucose-derived itaconate, α-ketoglutarate and 2-hydroxyglutarate levels without affecting succinate and citrate levels, indicating a partial inhibition of the TCA cycle. This indirect effect is associated with NRF2 (also known as NFE2L2) activation and anti-oxidant responses. These results suggest a moonlighting role of BCAT1 through redox-mediated control of mitochondrial function during early macrophage activation.

## Abstract (precis)

**Summary:** BCAT1 controls mitochondrial metabolism independently of its transamination role in activated human macrophages.

## INTRODUCTION

Toll-like receptor (TLR) activation by lipopolysaccharide (LPS) reprogrammes mononuclear phagocytes, which utilize glucose, fatty acids and amino acids in a coordinated way to mount a context-dependent immune response. Classically activated macrophages increase their glycolytic rate (Jha et al., 2015), undergo an early increase in tricarboxylic acid (TCA) cycle activity (Lauterbach et al., 2019; Seim et al., 2019) and mitochondrial reactive oxygen species (ROS) (Cameron et al., 2019), and progressively accumulate lactate. Hence, the temporally controlled TCA cycle and glycolysis-derived metabolites, together with ROS, participate collectively into early and late transcriptional and epigenetic regulation of pro-inflammatory macrophage polarization (Bambouskova et al., 2018; Lauterbach et al., 2019; Mills et al., 2016; Murphy and O'Neill, 2018; Zhang et al., 2019).

Amino acid availability is essential for efficient metabolic reprogramming of classically activated macrophages (Kelly and Pearce, 2020). Amino acid synthesis or usage can utilize shunts from glycolysis or the TCA cycle and participate in LPS-driven metabolic reprogramming. For instance, *de novo* serine biosynthesis is a glycolytic shunt required for inflammasome-independent production of IL-1β in LPS-stimulated macrophages (Rodriguez et al., 2019). Similarly, arginine is metabolized to nitric oxide (NO) and L-citrulline, which can intersect with the TCA cycle through the arginosuccinate shunt in classically activated macrophages (Jha et al., 2015). Furthermore, the anti-oxidant defence mechanisms, which are normally triggered to limit LPS-driven oxidative stress, use amino acids. The glutathione (GSH) pathway uses amino acids, such as cysteine, serine, glycine and methionine, in order to ensure cytoprotective responses against oxidative stress.

Amino acid catabolism can also feed into the TCA cycle and support ATP production through transamination reactions. These are responsible for the deamination of most amino acids and result in the formation of α-ketoacids, which could be used as precursors to enter the TCA cycle at α-ketoglutarate (αKG), succinyl-CoA, fumarate, oxaloacetate, pyruvate or acetyl-CoA. Essential amino acids comprise leucine, valine and isoleucine, also known as branched-chain amino acids (BCAAs) (Neinast et al., 2019). Their transamination is catalysed by BCAA transaminase (BCAT) enzymes encoded by cytoplasmic (BCAT1) and mitochondrial (BCAT2) genes (Sivanand and Vander Heiden, 2020). BCAAs can fuel the TCA cycle through transamination and production of glutamate and respective α-ketoacids that can undergo decarboxylation to provide succinyl-CoA (valine metabolism) or acetyl-CoA (leucine or isoleucine metabolism). The role of BCAAs in immunity has been studied in more detail through investigating leucine metabolism. Leucine can activate mammalian target of rapamycin complex 1 (mTORC1) (Hara et al., 1998; Sancak et al., 2008), and BCAT1 is an activator of mTORC1 (Ananieva et al., 2014; Martin et al., 2020). In macrophages, an uptake of leucine has been linked with LPS stimulation in RAW 264.7 cells (Meiser et al., 2016), and α-ketoisocaproate (KIC), the α-ketoacid derived from leucine transamination, was shown to inhibit macrophage phagocytosis (Silva et al., 2017). These studies suggest that both leucine uptake and BCAT1-mediated transamination are associated with macrophage function.

We have previously shown that BCAT1 is the most abundant isoform in human macrophages and its pharmacological inhibition by a leucine analogue (ERG240) is associated with decreased oxidative phosphorylation and with suppressed IRG1 (also known as ACOD1) and itaconate levels during early activation of macrophages with LPS (3–8 h) (Papathanassiu et al., 2017). ERG240 was anti-inflammatory when injected into the peritoneum of mice together with LPS, and it reduced sterile inflammation in crescentic glomerulonephritis and rheumatoid arthritis models (Papathanassiu et al., 2017). The effect of BCAT1 inhibition on the TCA cycle during early human macrophage activation suggested a link between the cytoplasmic BCAT1 function and mitochondrial TCA cycle activity, which leads to an overall anti-inflammatory phenotype. Hence, the exact role of leucine catabolism in the control of mitochondrial activity in human macrophages remains to be determined.

Here, we sought to determine the link between BCAT1 activity and mitochondrial function during early human macrophage polarization with LPS. We first show that glucose but not glutamine is utilised as part of the major anaplerotic pathway in these cells. Metabolic tracing experiments in conditioned media and cell extracts showed an early consumption of arginine and tryptophan, but the BCAA were not taken up, despite an increase in BCAT1 activity. We show an increase in TCA cycle volume upon early LPS stimulation in human macrophages. BCAT1 inhibition reversed this upregulation in TCA cycle activity by drastically reducing glucose-derived itaconate, αKG, glutamate and 2-hydroxyglutarate (2-HG) levels. We show that leucine is not catabolized by BCAT1 to integrate into the TCA cycle in basal or LPS-stimulated macrophages. Interestingly, BCAT1 inhibition triggers an NRF2 (NFE2L2)-mediated anti-oxidant response, indicating a possible moonlighting role of this enzyme through redox-mediated control of mitochondrial function during early macrophage activation.

## RESULTS

### Glucose drives the early increase in TCA cycle activity in pro-inflammatory human macrophages

Glucose and glutamine are major energy sources that control macrophage function, but their relative contribution to the early phase of macrophage activation in humans is not well described. Using stable isotope tracing followed by liquid chromatography-mass spectrometry (LC-MS), we thus measured the contribution of glucose and glutamine to TCA cycle activity in human monocyte-derived macrophages (hMDMs) isolated from healthy donors during early activation.

**Fig. 1.**
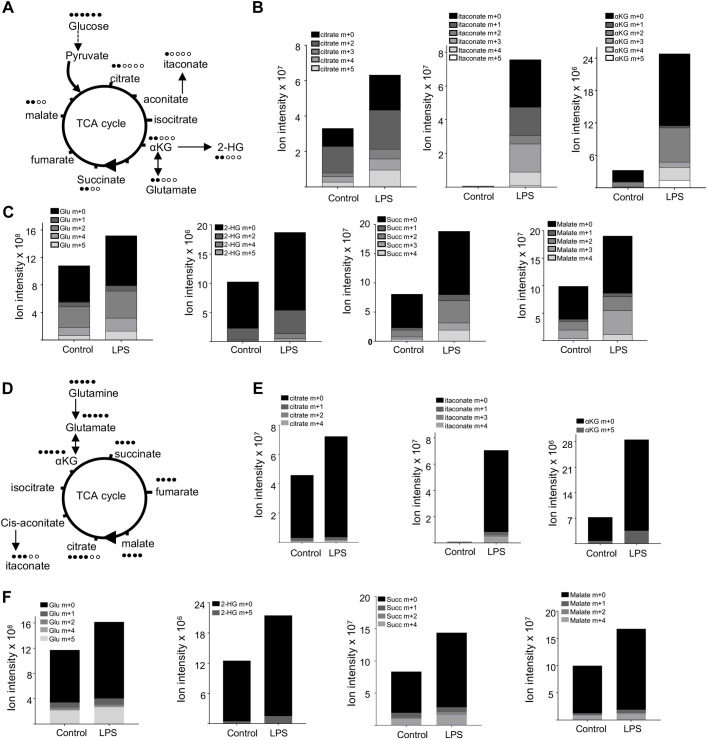
**Glucose but not glutamine is a major carbon source for TCA cycle metabolites during early human macrophage activation.** (A) Diagram of uniformly labelled [U-^13^C]-glucose catabolism, highlighting the TCA cycle metabolites and their expected glucose-derived ^13^C atoms (filled circles). (B,C) Glucose-derived and M+0 metabolites measured by LC-MS in control and LPS (8 h; 100 ng/ml)-stimulated hMDMs; *n*=6 donors. See Fig. S1 for individual data points. Glu, glutamate; Succ, succinate. (D) Diagram of uniformly labelled [U-^13^C]-glutamine catabolism, highlighting the TCA cycle metabolites and their expected glutamine-derived ^13^C atoms (filled circles). (E,F) Glutamine-derived and M+0 metabolites measured by LC-MS in control and LPS (8 h; 100 ng/ml)-stimulated hMDMs; *n*=6 donors. See Fig. S1 for individual data points.

In the presence of uniformly labelled [U-^13^C]-glucose, incubation with LPS for 8 h resulted in an upregulation of glucose-derived itaconate (M+1, single ^13^C-labelled itaconate), as well as an overall increase in TCA cycle and αKG-derived metabolites (Fig. 1A–C; Fig. S1).

To test whether basal activity or early activation with LPS rely on glutamine usage, we next used uniformly labelled [U-^13^C]-glutamine and followed its potential incorporation into the TCA cycle in human macrophages (Fig. 1D). Incubation with [U-^13^C]-glutamine led to ^13^C-labelled itaconate, TCA cycle and αKG-derived metabolites (Fig. 1D–F; Fig. S1). However, glucose catabolism contributed significantly more than glutamine to citrate, itaconate, αKG, glutamate, 2-HG, succinate and malate levels upon early LPS stimulation (Fig. S2). Taken together, these results showed that glucose but not glutamine is a major source for the increased TCA cycle volume during early macrophage activation with LPS.

### Short-term LPS exposure increases leucine transamination without inducing BCAA uptake and catabolism

**Fig. 2.**
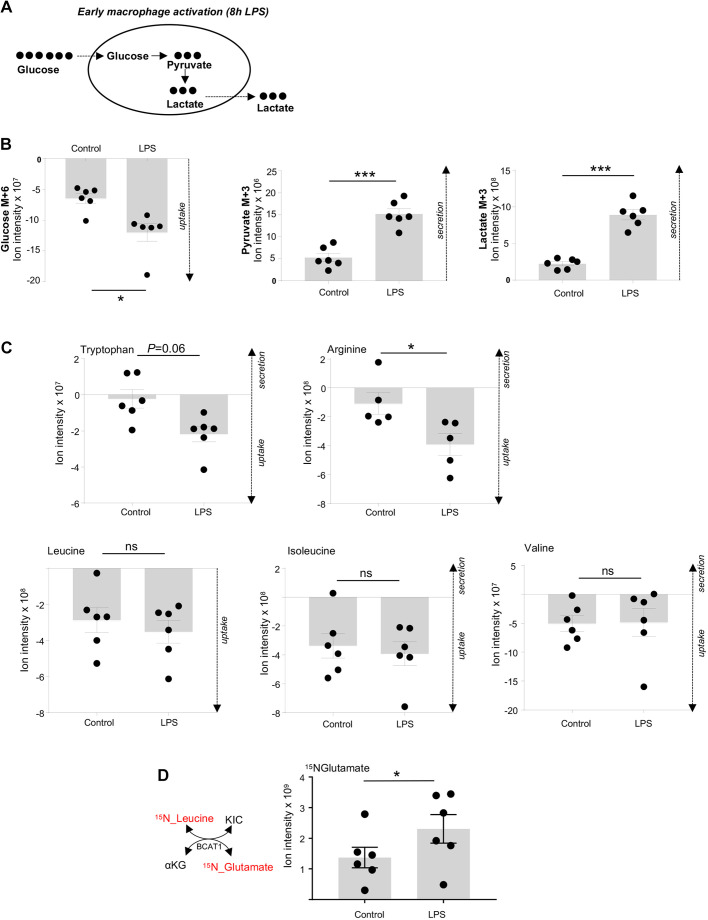
**Short-term LPS exposure increases leucine transamination without inducing BCAA uptake and catabolism.** (A) Schematic of glucose-derived pyruvate and lactate adducts in macrophages treated with uniformly labelled [U-^13^C]-glucose. Filled circles indicate glucose-derived ^13^C atoms. (B) Uptake and secretion profiles by LC-MS shown for M+6 glucose, M+3 pyruvate and M+3 lactate in basal (control) and LPS (8 h; 100 ng/ml)-stimulated hMDMs. (C) Tryptophan, arginine and BCAA uptake in basal (control) and LPS (8 h; 100 ng/ml)-stimulated hMDMs. (D) Leucine transamination by BCAT1 (left) and [^15^N]-glutamate amount measured by LC-MS in hMDMs incubated with [^15^N]-leucine in control and LPS (8 h; 100 ng/ml)-stimulated hMDMs. Mean±s.e.m and individual data points are shown for at least *n*=5 donors. *P<0.05; ***P<0.001; ns, not significant (paired *t*-test).

**Fig. 3.**
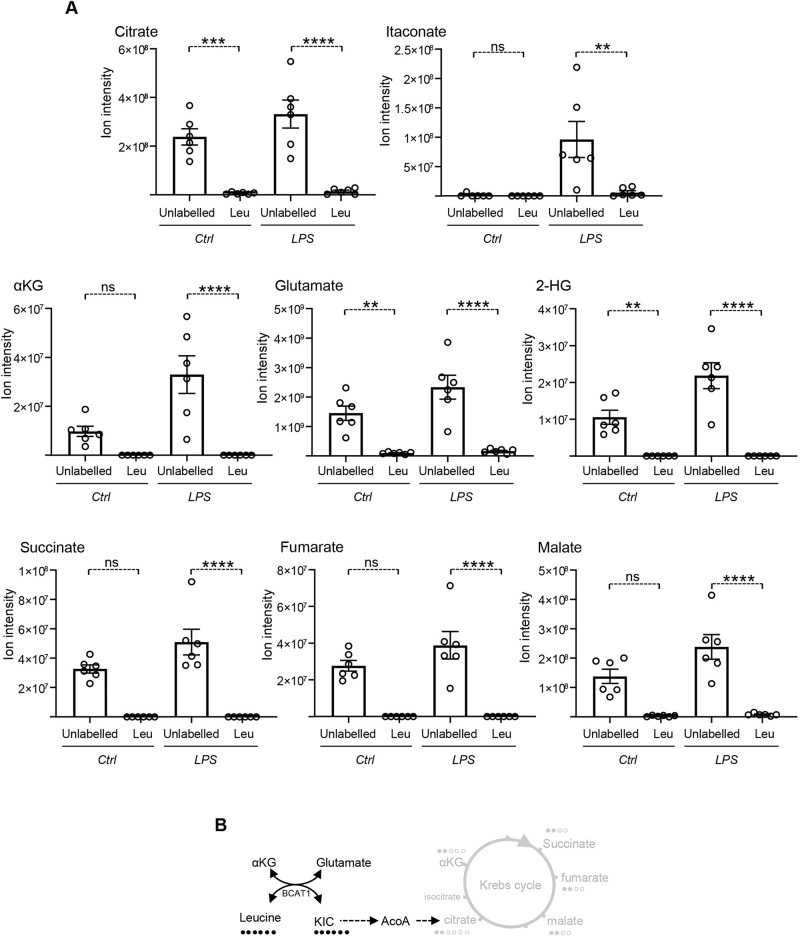
**Leucine is not metabolized to integrate into the TCA cycle in basal or stimulated human macrophages.** (A) LC-MS for unlabelled and [U-13C]-leucine-derived (Leu) metabolites in control (Ctrl) and LPS (100 ng/ml; 8 h)-treated hMDMs. (B) Diagram of uniformly labelled [U-^13^C]-leucine catabolism, highlighting the possibly entry to the TCA cycle through acetyl-coA (AcoA). The TCA cycle is shown in grey to indicate there were no leucine-derived ^13^C atoms (filled circles) found in any of the metabolites. Mean±s.e.m and individual data points are shown for at least *n*=5 donors. **P<0.01; ***P<0.001; ****P<0.0001; ns, not significant (one-way ANOVA followed by Tukey's test).

The increased TCA cycle activity upon early LPS stimulation in human macrophages suggested that these cells may use extracellular amino acids (in particular BCAAs) as energy sources additional to glucose, in order to support the rise in mitochondrial activity. To test this hypothesis, we incubated hMDMs from healthy donors with uniformly labelled [U-^13^C]-glucose and evaluated the uptake or secretion by measuring different metabolites in the conditioned culture media (CCM) and cell extracts. We first confirmed that M+6 glucose uptake is increased upon 8 h LPS stimulation and that glucose-derived pyruvate (M+3 pyruvate) and lactate (M+3 lactate) accumulate rapidly in the CCM (Fig. 2A,B). Early LPS stimulation did also result in an increase in the uptake of tryptophan and arginine, whereas BCAA levels did not differ between the cell extracts and the CCM in hMDMs (Fig. 2C). Although BCAA uptake was not induced by early LPS stimulation, the transamination of leucine, measured by quantifying [^15^N]-glutamate in hMDMs incubated with [^15^N]-leucine, showed increased BCAT1 activity (Fig. 2D). To test whether leucine–KIC–acetyl-CoA is a possible anaplerotic pathway, we incubated hMDMs with [U-^13^C]-leucine, and followed its fate in basal and LPS-stimulated human macrophages. We found negligible contribution of leucine-derived ^13^C atoms to the measured TCA cycle intermediates (i.e. citrate, αKG, succinate, fumarate and malate) in basal or stimulated macrophages (Fig. 3A). This indicates that leucine is not fully oxidized in the mitochondria of human macrophages (Fig. 3B).

### BCAT1 inhibition decreases TCA cycle activity independently of leucine transamination

**Fig. 4.**
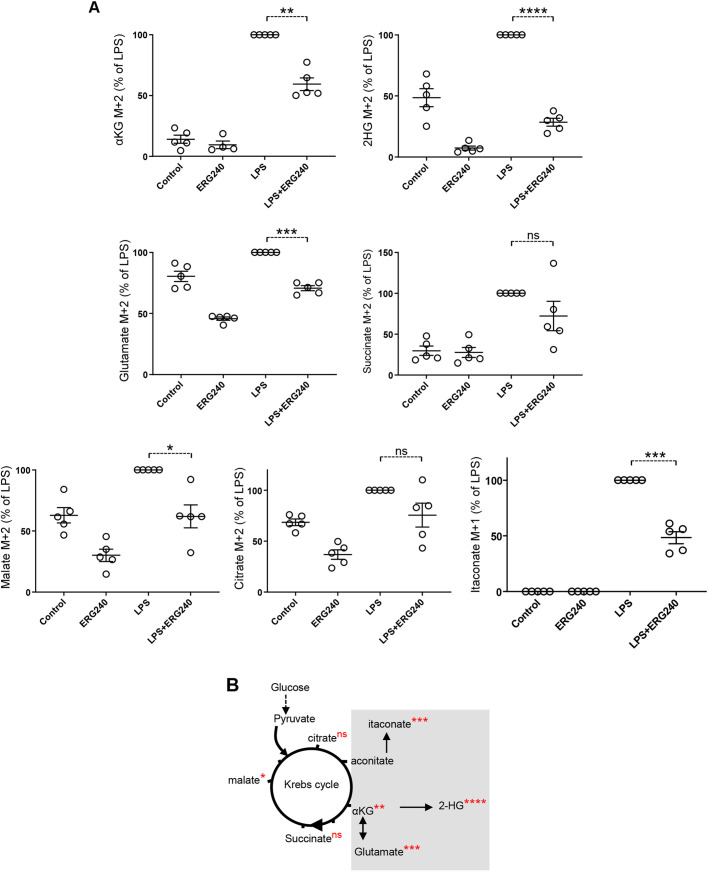
**BCAT1 inhibition results in partial inhibition of the TCA cycle between citrate and succinate.** (A) Glucose-derived isotopologues (citrate M+2, itaconate M+1, αKG M+2, glutamate M+2, 2-HG M+2, succinate M+2, malate M+2) levels were measured in control, ERG240-treated, LPS-stimulated (8 h; 100 ng/ml) and LPS-stimulated and ERG240-treated (8 h; LPS+ERG240) hMDMs. (B) Diagram of the TCA cycle metabolites and their derivative metabolites, highlighting the reactions affected by BCAT1 inhibition (grey box). For each metabolite, the statistical significance of the effect of BCAT1 inhibition is shown. Mean±s.e.m and individual data points are shown for *n*=5 donors. *P<0.05; **P<0.01; ***P<0.001; ****P<0.0001; ns, not significant (one sample *t*-test).

We have previously shown that BCAT1 inhibition results in reduced *IRG1* mRNA and protein levels and decreased itaconate levels during early activation of human macrophages (Papathanassiu et al., 2017). In order to have a comprehensive measurement of the effect of BCAT1 on mitochondrial activity, we next measured itaconate, TCA cycle and αKG-derived metabolites in hMDMs following BCAT1 inhibition. The leucine analogue ERG240 inhibits BCAT1 transamination activity and completely rescues its upregulation upon LPS stimulation, leading to a transient intracellular accumulation of leucine (Fig. S3). By incubating human macrophages with ERG240, we showed decreased glucose-derived M+1 itaconate (Fig. 4A). Interestingly, BCAT1 inhibition was associated with marked reductions in specific metabolites derived from TCA cycle activity, such as M+2 αKG, M+2 glutamate and M+2 2-HG, whereas M+2 succinate and M+2 citrate levels were not significantly affected (Fig. 4A). Although BCAT1 inhibition had no apparent effect on the unlabelled pool of these metabolites, there was a significant effect on mostly glucose-derived itaconate, αKG and glutamate isotopologues (Fig. S4). Furthermore, glucose-derived pyruvate and lactate levels were not affected by BCAT1 inhibition in activated human macrophages (Fig. S5). These results indicated that BCAT1 inhibition primarily affects metabolites positioned between citrate and succinate within the TCA cycle in human macrophages (Fig. 4B).

### Inhibition of BCAT1 activity activates NRF2 and triggers anti-oxidant responses

**Fig. 5.**
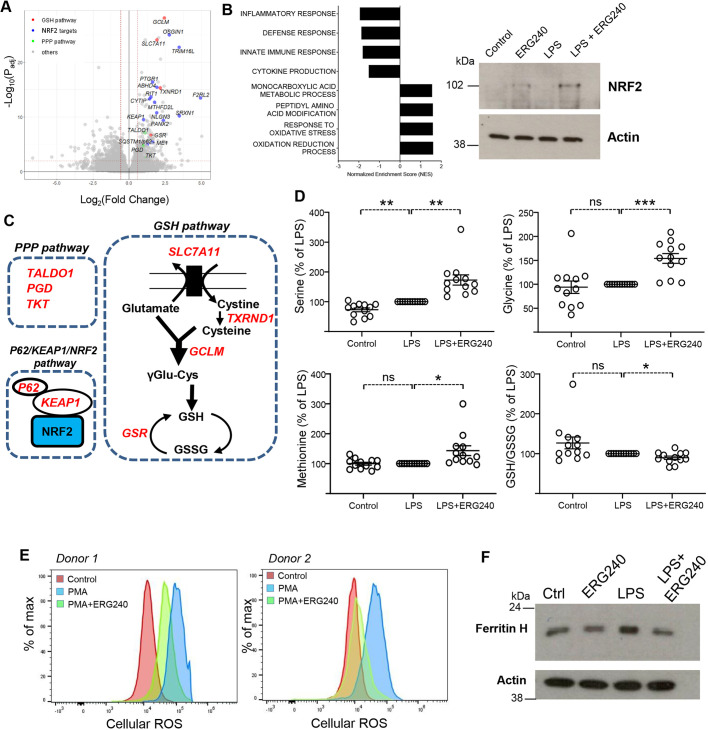
**BCAT1 inhibition activates NRF2 and is anti-oxidant in human macrophages.** (A) Volcano plot for RNA-seq analysis of hMDMs treated with LPS (8 h; 100 ng/ml) or LPS and ERG240 (8 h; LPS+ERG240); *n*=3 donors. The dotted lines show the thresholds used to define differentially expressed genes (fold change<1.5, fold change>1.5; *P*_adj_<0.01). (B) Gene Set Enrichment Analysis (GSEA) for RNA-seq analysis (left panel; LPS versus LPS+ERG240) and NRF2 western blotting (right panel) in hMDMs. Actin is shown as a loading control. (C) Schematic representation of the pathways affected in the RNA-seq analysis. The genes in red are upregulated in LPS+ERG240 when compared to LPS in hMDMs. (D) LC-MS quantification of serine, glycine, methionine and GSH/glutathione disulfide (GSSG) in basal (control), LPS-treated (8 h; 100 ng/ml) and LPS+ERG240-treated hMDMs. Mean±s.e.m and individual data points are shown for *n*=12 donors. (E) Flow cytometry analysis of live hMDMs (*n*=2 donors). Mean fluorescence intensity (MFI) was quantified as a measure of cellular ROS production (Cell ROX) in control, PMA only (PMA) and ERG240 pre-treated (ERG240+PMA) cells. ERG240 pre-treatment was for 16 h; PMA stimulation was for 30 min. (F) Ferritin heavy chain (Ferritin H) western blotting in control (Ctrl), ERG240, LPS (16 h; 100 ng/ml) and LPS+ERG240 hMDMs. Actin is shown as a loading control. *P<0.05; **P<0.01; ***P<0.001; ns, not significant (one sample *t*-test).

In order to identify pathways that link BCAT1 activity to the control of TCA cycle activity in mitochondria, we next performed RNA sequencing (RNA-seq) in human macrophages stimulated with LPS (8 h) in presence or absence of the BCAT1 inhibitor. Interestingly, the top upregulated gene following BCAT1 inhibition was *GCLM* [log_2_ fold change of 2.43, Benjamini–Hochberg adjusted *P* (*P*_adj_)=8.56×10^−29^], which encodes the rate-limiting enzyme in GSH synthesis (Fig. 5A). A closer look at the set of the most significantly upregulated transcripts revealed genes belonging to oxidative stress response pathways, namely GSH and NRF2 (Fig. 5A,B), whereas inflammatory and innate immune response pathways were downregulated (Fig. 5B). The NRF2–KEAP1 protein complex acts a cellular redox sensor and maintains redox homeostasis by regulating the transcription of anti-oxidant genes involved in GSH synthesis and reduction. The latter is dependent on NADPH produced by the pentose phosphate pathway (PPP) enzymes, which are NRF2 targets (Fig. 5C). Genes belonging to PPP and GSH pathways, as well as those that interact with NRF2 or the NRF2 complex (*KEAP1*, *SQSTM1*) were significantly upregulated upon BCAT1 inhibition (Fig. 5C). We confirmed enhanced NRF2 protein levels upon BCAT1 inhibition in LPS-stimulated hMDMs (Fig. 5B) and showed that BCAT1 inhibition and silencing upregulates NQO1 protein levels and mRNA of NRF2 target genes, respectively (Fig. S5). Interestingly, a brief exposure (30 min) to the BCAT1 inhibitor induced ∼50% rise in mitochondrial ROS (Fig. S5). To further strengthen evidence for the induction of the GSH pathway upon BCAT1 inhibition, we performed LC-MS analysis and showed a general increase in the metabolites of this pathway in LPS and ERG240 co-treated human macrophages (Fig. 5D). In keeping with the induction of the NRF2-mediated anti-oxidant response, BCAT1 inhibition reduced NADPH oxidase (NOX)-derived ROS in activated human macrophages (Fig. 5E). We also used ferritin levels as an additional readout of intracellular oxidative stress, as previously established (Orino et al., 2001). ERG240 reduced LPS-induced ferritin accumulation (Fig. 5F), confirming the overall anti-oxidant response due to BCAT1 inhibition.

## DISCUSSION

In this study, we describe the metabolic rewiring following early activation of human macrophages with LPS and focus on leucine metabolism and its regulation through BCAT1 activity. As expected, we have shown that glucose is rapidly taken up by human macrophages stimulated for 8 h with LPS and used in glycolysis. This early activation period is also characterized by a general increase in TCA cycle activity, which preferentially uses glucose-derived substrates. Glutaminolysis is a pathway that uses hydrolysis of glutamine to glutamate, which can then integrate into the TCA cycle through αKG. Thus, both glucose and glutamine are energy sources and initiate mitochondria-derived signalling events in activated macrophages (Martinez-Reyes and Chandel, 2020; van Teijlingen Bakker and Pearce, 2020). Glutamine has been previously shown to be utilized by M1, M2 and phospholipid-exposed M1 murine macrophages (Di Gioia et al., 2020; Jha et al., 2015; Palmieri et al., 2020; Tannahill et al., 2013; Wallace and Keast, 1992). Furthermore, macrophages can also secrete glutamine and participate in muscle regeneration (Shang et al., 2020). Here we report that glutamine is not a major contributor to TCA cycle activity during the early phase of human macrophage activation with LPS, which suggests that glutamine-dependent metabolic pathways are time-, context- and species-dependent. In line with this, a recent elegant study showed that glutamine-based anaplerosis is a compensatory response to nitric oxide-mediated inhibition of the TCA cycle in murine macrophages (Palmieri et al., 2020). Inflammatory human macrophages produce very little NO *in vitro* (Weinberg et al., 1995), and, unlike mice, human macrophages do not show a robust upregulation of *NOS2* upon LPS stimulation, because of epigenetic silencing of the human gene (Gross et al., 2014). Thus the lack of glutamine-based anaplerosis in classically activated human macrophages could be attributed to the absence of robust NO production or an *NOS2* response in these cells.

Early LPS stimulation is also characterized by an uptake of tryptophan and arginine, two amino acids that are described within the context of macrophage polarization through their respective key metabolic enzymes: indoleamine 2,3-dioxygenase 1 (IDO1) for tryptophan and arginase 1 (ARG1) for arginine (Saha et al., 2017; Viola et al., 2019). Although the expression of ARG1 has been associated with alternative macrophage activation, the uptake of arginine increases upon 8 h LPS stimulation, in line with the initial burst in NO sustained by arginine uptake from the medium in activated macrophages (Mori and Gotoh, 2000). In keeping with our results, 70–80% of tryptophan has been reported to be depleted from cell culture supernatants following 24 h LPS stimulation in human macrophages (Regan et al., 2018). Interestingly, sequential activation of ARG1 and IDO1 promotes a potent immunoregulatory phenotype in conventional dendritic cells, highlighting an interplay between tryptophan and arginine metabolisms (Mondanelli et al., 2017). Thus, the interplay between tryptophan and arginine metabolisms could be a feature of early LPS stimulation in human macrophages. We report no increase in BCAA uptake during the early activation period of human macrophages, despite an increase in BCAT1 activity. Because leucine does not participate in the increased LPS-driven TCA cycle activity in human macrophages, the increase in BCAT1 activity can therefore reflect an alternative function of this enzyme. The absence of the contribution of leucine to the TCA cycle is consistent with a previous report where the authors investigated the fate of leucine-derived 1,2-^13^C_2_-KIC in human macrophages (Silva et al., 2017). In accordance with our findings, the authors did not find any labelled TCA cycle intermediate, further supporting an alternative usage of BCAT1 activity during early macrophage activation in humans.

BCAT1 inhibition results in a partial inhibition of the TCA cycle associated with an oxidative stress response driven by NRF2. Our study presents two limitations. First, the molecular origin of the anti-oxidant responses triggered by the inhibition of BCAT1 remains to be identified. Second, the mechanisms through which the NRF2-mediated anti-oxidant responses are linked to TCA cycle activity, specifically between citrate and succinate, warrant further investigation. BCAT1 is a redox-acting protein that contains thiol residues allowing oxidation, S-nitrosylation and S-glutathionylation reactions, which are possibly responsible for its moonlighting role (Conway et al., 2008; Conway and Lee, 2015; Harris et al., 2020; Hindy and Conway, 2019). Subsequently, BCAT1 inhibition *per se*, rather than secondary effects on GSH biosynthesis (McBrayer et al., 2018), could initiate an oxidative stress that triggers an anti-oxidant response. In keeping with this, we have observed that BCAT1 inhibition rapidly induced mitochondrial ROS (within 30 min) in human macrophages. Although the link between cytoplasmic leucine metabolism and mitochondrial function is intriguing, mitochondrial ROS has been recently shown to activate NRF2 in macrophages (Wang et al., 2019b) and could explain the reduction of TCA cycle activity upon BCAT1 inhibition. Indeed, among the TCA cycle enzymatic steps influenced by BCAT1, the citrate–isocitrate interconversion is catalysed by mitochondrial aconitase (ACO2), whose activity is tightly dependent on ROS levels. Under oxidative stress, aconitase iron–sulfur clusters participate in a Fenton-type reaction and lose their activity (Chang et al., 2016; Gardner et al., 1994), and acute iron deprivation blocks mitochondrial aconitase activity and TCA cycle activity in human macrophages (Pereira et al., 2019).

In summary, a rapid mitochondrial oxidative stress generated by BCAT1 inhibition is likely to cause the NRF2 response and the downregulation of metabolites positioned between citrate and succinate through the inhibition of ACO2 activity. NRF2 induction is anti-inflammatory in mouse macrophages (Kobayashi et al., 2016; Mills et al., 2018), and peritoneal injection of ERG240 in mice also confers a largely anti-inflammatory phenotype, supporting the existence of an anti-inflammatory BCAT1–NRF2 pathway. Further studies will have to establish the exact immunomodulatory effects of NRF2 inhibition in human macrophages in order to study the NRF2 pathway in the context of BCAT1 inhibition. Our proposed mechanistic link assumes an activation of NRF2, independently of itaconate, a metabolite which is linked to BCAT1 activity (Papathanassiu et al., 2017), capable of inducing NRF2 in activated macrophages (Mills et al., 2018). In fact, recent reports have shown that natural endogenous itaconate behaves differently from electrophilic derivatives such as 4-octyl itaconate (Sun et al., 2020a; Swain et al., 2020). Swain et al. (2020) showed that endogenous itaconate is not a robust NRF2 inducer, which supports the assumption that BCAT1-mediated NRF2 induction is independent of itaconate and may arise from mitochondrial ROS in activated human macrophages.

A crosstalk between leucine metabolism and the mitochondria-derived itaconate has been proposed in *Mycobacterium tuberculosis* through the identification of a novel bifunctional enzyme involved in itaconate dissimilation and L-leucine catabolism (Wang et al., 2019a). Furthermore, a possible moonlighting role of BCAT enzymes has been previously suggested in yeast. Kingsbury et al. (2015) found that yeast mitochondrial branched-chain amino acid transferase Bat1 interacts with mitochondrial aconitase and controls TCA cycle activity and TORC1 signalling independently of its catalytic function; a finding that endorses an association between cytoplasmic leucine metabolism and mitochondrial function in macrophages. In keeping with this, a screening assay in yeast identified non-catalytic functions for the cytosolic branched-chain amino acid transferase Bat2 (Espinosa-Cantu et al., 2018), and the presence of a redox-active CXXC motif in BCAT enzymes has been linked to a possible moonlighting role (Conway, 2020; Conway and Lee, 2015) that could involve the regulation of redox homeostasis. Indeed, human peripheral blood mononuclear cells incubated with high concentration of BCAAs (a scenario equivalent to BCAT inhibition) show elevated production of ROS (Zhenyukh et al., 2017), and *Caenorhabditis elegans bcat-1* knockdown induces oxidative damage in neurons (Mor et al., 2020). It is also important to consider species- and stimuli-specific differences in the generation of oxidative stress because, unlike PMA, LPS is not a strong inducer of ROS in human monocytes (Bulua et al., 2011). This is particularly important in the context of immunomodulatoy effects of NRF2 induction, which can be distinct in human macrophages when compared to mouse cells.

Growing evidence shows that leucine transport and/or metabolism affect macrophage function (Grajeda-Iglesias et al., 2018; Meiser et al., 2016; Papathanassiu et al., 2017; Silva et al., 2017; Wuggenig et al., 2020; Yoon et al., 2018). Therefore leucine degradation through BCAT1 coupled with redox homeostasis and the control of mitochondrial function may provide a new angle to study macrophage immunometabolism. Furthermore, the *in vivo* consequences of the results presented in this study can be evaluated within the skeletal homeostasis context. We have recently shown that *Bcat1*, as part of a network enriched for mTORC1 genes, is essential for osteoclast multinucleation, and its deletion is associated with increased bone mass due to an osteoclast-related effect (Kang et al., 2014; Pereira et al., 2020). Osteoclasts are metabolically active and mitochondria-rich cells, and it is therefore likely that BCAT1–NRF2-mediated mitochondrial activity is essential for osteoclast formation. A recent report corroborates this hypothesis (Sun et al., 2020b).

In conclusion, we show a possible moonlighting role of BCAT1 in early macrophage activation. Our results suggest that BCAT1 inhibition could be used as a therapeutic way of inducing anti-oxidant and anti-inflammatory responses in macrophage-dependent inflammatory disease.

## MATERIALS AND METHODS

### Reagents

The antibodies used in western blot analyses were as follows: anti-NRF2 (1:1000; D1Z9C; #12721; Cell Signaling Technology), anti-ferritin H (1:1000; heavy chain antibody; Y-16; sc14416; Santa Cruz Biotechnology), anti-ACTB [1:2000; β-Actin (C4); sc-47778; Santa Cruz Biotechnology]. Lipopolysaccharide (LPS; *Escherichia coli* serotype O111:B4; Cat No L4391; 100 ng/ml) and phorbol 12-myristate 13-acetate (PMA; Cat No P1585; 1 µM) were purchased from Sigma. ERG240 was produced by Ergon Pharmaceuticals and used at 20 mM.

### hMDM culture and stimulation

Human monocyte-derived macrophages (hMDMs) were differentiated from de-identified buffy cones from anonymous healthy donors (no identifiable private information) using gradient separation (Histopaque 1077, Sigma) and adhesion purification. Following Histopaque separation, peripheral blood mononuclear cells were resuspended in RPMI (Life Technologies), and monocytes were purified by adherence for 1 h at 37°C and 5% CO_2_. The monolayer was washed three times with HBSS (Invitrogen) to remove non-adherent cells, and monocytes were matured for 5 d in RPMI containing 100 ng/ml macrophage colony-stimulating factor (M-CSF; PeproTech, London, UK) and 10% foetal calf serum (Labtech International)*.* hMDMs were treated with LPS (100 ng/ml, 8 h) or with LPS and ERG240 (20 mM, 8 h) unless otherwise stated in the figure legends.

### RNA extraction, library preparation and data analysis

Total RNA was extracted from hMDMs using Trizol (Invitrogen) and an RNeasy mini kit (Qiagen) according to the manufacturer's instructions, with an additional purification step by on-column DNase treatment using an RNase-free DNase Kit (Qiagen) to ensure elimination of any genomic DNA. The integrity and quantity of total RNA was determined using a NanoDrop 1000 spectrophotometer (Thermo Fisher Scientific) and Agilent 2100 Bioanalyzer (Agilent Technologies). Total RNA (500 ng) was used to generate RNA-seq libraries using an NEBNext Ultra II Directional RNA Library Prep kit for Illumina, according to the manufacturer's instructions. Briefly, RNA was purified and fragmented using poly-T oligo-attached magnetic beads using two rounds of purification followed by the first and second cDNA strand synthesis. Next, cDNA 3′ ends were adenylated and adapters ligated followed by 11 cycles of library amplification. The libraries were size selected using AMPure XP Beads (Beckman Coulter), purified, and their quality was checked using an Agilent 2100 Bioanalyzer. Samples were randomized to avoid batch effects, and multiplexed libraries were run on a single lane (eight samples per lane) of a HiSeq 2500 platform (Illumina) to generate 100-bp paired-end reads.

An average depth of 50.6 M reads per sample was achieved. Sequencing adapters were removed using Trimmomatic (v.0.36) and the read quality was checked using FastQC (v.0.11.2) before and after trimming. Reads were aligned to the human genome (GRCh38.primary_assembly.genome.fa; annotation: gencode.v25.annotation.gtf) using the tophat2 package (v.2.1.0: -b2-sensitive,--library-type fr-firststrand). The average mapping percentage of 97% was achieved, and the average number of properly paired reads was 48.7 M (∼92%). Mapping quality, read distribution, gene body coverage, GC content and rRNA contamination were checked using picard (v.2.6.0) software. Gene level read counts were computed using HT-Seq-count (v.0.6.1, annotation: gencode.v25.annotation.gtf) in strict ‘-m intersection-strict’ mode. Differential gene expression analysis between groups was performed using DESeq2 (v.1.14.1), and significantly differentially expressed genes were reported using a threshold of fold change at 1.5× and a Benjamini–Hochberg adjusted *P*-value (*P*_adj_) below 1%. Volcano plots of differentially expressed genes were generated using ggplot2 (v.3.0.0; https://cran.r-project.org/web/packages/ggplot2/index.html) package. All raw RNA-seq data processing steps were performed in the Cx1 high-performance cluster computing environment, Imperial College London. Further analyses were conducted in R/Bioconductor environment v.3.4.4 (https://www.r-project.org/). The human macrophage RNA-seq data has been deposited in the NCBI's Gene Expression Omnibus database (GEO GSE148701).

Gene Set Enrichment Analysis (GSEA v5.2; http://software.broadinstitute.org/gsea/index.jsp) was utilized to further assess whether specific biological pathways or signatures were significantly enriched between different treatment groups. GSEA determines whether an *a priori* defined gene set shows statistically significant cumulative changes in gene expression between phenotypic subgroups. In brief, all differentially expressed genes were ranked according to the fold change of expression between two groups. Next, an enrichment score was calculated for a given gene set based on its position in the ranked differential list. One thousand random permutations of the phenotypic subgroups were used to establish a null distribution of enrichment score against which a normalized enrichment score (NES) and false discovery rate (FDR)-corrected *q* values were calculated. All gene sets with an FDR less than 0.25 were considered as statistically significant.

### Stable isotope tracing by liquid chromatography-mass spectrometry

hMDMs were incubated for 8 h with SILAC medium (GIBCO, A2494201; lacking glucose and Phenol Red) with addition of L-glutamine (0.5 mM), arginine (200 mg/l), lysine (40 mg/l) and uniformly labelled glucose (2 g/l D-Glucose U-^13^C, 99%; Cambridge isotope laboratories). For labelled glutamine experiments, the SILAC medium was supplemented with L-glutamine (^13^C_5_, 99%; Cambridge Biosciences). For labelled leucine experiments, leucine-deprived RPMI1640 (R1780, Sigma) was supplemented with arginine and lysine and with either [^15^N]-leucine (98%; Cambridge Biosciences) or [U-^13^C]-leucine (99%; Cambridge Biosciences).

Cells were then washed three times with phosphate-buffered saline (PBS) and 200 µl of extraction buffer (50% LC-MS grade methanol, 30% acetonitrile and 20% ultrapure water) was added per 10^6^ cells. Following 15 min incubation in dry ice, the cells were scraped off and kept under vigorous shaking for 15 min at 4°C, then left for 1 h incubation at −20°C. Following centrifugation, the supernatant was stored at −80°C until further analysis. For the cell supernatant analysis, the same procedure was performed with supernatant obtained from 10^6^ cells.

Samples were randomized in order to avoid bias due to machine drift and were processed blindly. LC-MS analysis was performed using a Q Exactive mass spectrometer coupled to a U3000 UHPLC system (Thermo Fisher Scientific). The liquid chromatography system was fitted with a Sequant ZIC-pHILIC column (150 mm×2.1 mm) and guard column (20 mm×2.1 mm) from Merck Millipore (Germany) and temperature maintained at 40°C. The mobile phase was composed of 20 mM ammonium carbonate and 0.1% ammonium hydroxide in water (solvent A), and acetonitrile (solvent B). The flow rate was set at 200 µl/min, with the gradient as described previously (Mackay et al., 2015). The mass spectrometer was operated in full MS and polarity switching mode. The acquired spectra were analysed using XCalibur Qual Browser and XCalibur Quan Browser software (Thermo Fisher Scientific).

### Analysis of proteins

hMDMs were lysed in Laemmli sample buffer supplemented with protease inhibitors [Thermofisher, Halt™ Protease Inhibitor Cocktail (100×)] and resolved by SDS-PAGE, transferred onto PVDF membranes, then subjected to immunoblotting with the primary antibodies described above and secondary detection antibodies. The probed proteins were detected using SuperSignal West Femto chemiluminescent substrate (Thermo Fisher Scientific, Rockford, IL, USA).

### RNA interference

Human macrophages were re-plated in six-well plates (1×10^6^ cells per well) in RPMI (Invitrogen) overnight and transfected with siGENOME SMARTpool for human BCAT1 (100 nM, Dharmacon SMART pool) or non-targeting siRNA pool, as the scrambled control siRNA, using Dharmafect 1 (1:50, Dharmacon) as a transfection reagent in OPTIMEM medium (Invitrogen).

### Quantitative reverse transcription PCR

Total RNA was extracted from human macrophages using TRIzol reagent (Invitrogen), according to the manufacturer's instructions, and cDNA was synthesized using an iScript cDNA Synthesis kit (Bio-Rad). A total of 5 ng cDNA for each sample was used. All quantitative PCRs were performed on a ViaA 7 Real-Time PCR System (Life Technologies) using Brilliant II SYBR Green QPCR Master Mix (Agilent), followed by ViiA 7 RUO Software for the determination of Ct values. Results were analysed using the comparative Ct method, and each sample was normalized to the reference mRNA level of the *HPRT* gene, to account for any potential cDNA loading differences. Primer sequences are available upon request.

### Cellular and mitochondrial ROS

Cultured hMDMs were washed twice with PBS and incubated with CellROX Deep Red reagent (Invitrogen, C10422) or MitoSOX Red reagent (Invitrogen, M36008) at 5 μM for 30 min at 37°C to detect intracellular oxidative stress. hMDMs were harvested using cell dissociation buffer (Sigma), then washed and stained with LIVE/DEAD Fixable Aqua (Invitrogen, L34957) for 10 min without light before being resuspended in PBS containing 1% BSA and assayed using a LSR Fortessa Flow cytometer (BD Biosciences). Data were analysed using FlowJo software, version 7.6.5 (TreeStar Inc, Ashland, OR, USA). PMA (1 µm) was added to the cells for 30 min before acquiring the mean fluorescence intensity (MFI). ERG240 pre-treatment was for 16 h.

### Statistical analyses

Data are presented as mean±s.e.m. and analysed using GraphPad Prism software (version 7.02; GraphPad). Differences in LC-MS metabolites were tested by paired *t*-test or one-way ANOVA followed by a post-test as indicated. One sample t-tests were used when mean values were compared to the reference value of 100%. For the statistical analysis of the RNA-seq data, please refer to the RNA extraction, library preparation and data analysis section above.

## Supplementary Material

Supplementary information

Reviewer comments

## References

[JCS247957C1] Ananieva E. A., Patel C. H., Drake C. H., Powell J. D. and Hutson S. M. (2014). Cytosolic branched chain aminotransferase (BCATc) regulates mTORC1 signaling and glycolytic metabolism in CD4^+^ T cells. *J. Biol. Chem.* 289, 18793-18804. 10.1074/jbc.M114.554113 24847056 PMC4081922

[JCS247957C2] Bambouskova M., Gorvel L., Lampropoulou V., Sergushichev A., Loginicheva E., Johnson K., Korenfeld D., Mathyer M. E., Kim H., Huang L.-H. et al. (2018). Electrophilic properties of itaconate and derivatives regulate the IκBζ-ATF3 inflammatory axis. *Nature* 556, 501-504. 10.1038/s41586-018-0052-z 29670287 PMC6037913

[JCS247957C3] Bulua A. C., Simon A., Maddipati R., Pelletier M., Park H., Kim K.-Y., Sack M. N., Kastner D. L. and Siegel R. M. (2011). Mitochondrial reactive oxygen species promote production of proinflammatory cytokines and are elevated in TNFR1-associated periodic syndrome (TRAPS). *J. Exp. Med.* 208, 519-533. 10.1084/jem.20102049 21282379 PMC3058571

[JCS247957C4] Cameron A. M., Castoldi A., Sanin D. E., Flachsmann L. J., Field C. S., Puleston D. J., Kyle R. L., Patterson A. E., Hässler F., Buescher J. M. et al. (2019). Inflammatory macrophage dependence on NAD^+^ salvage is a consequence of reactive oxygen species-mediated DNA damage. *Nat. Immunol.* 20, 420-432. 10.1038/s41590-019-0336-y 30858618 PMC12842115

[JCS247957C5] Chang H.-C., Wu R., Shang M., Sato T., Chen C., Shapiro J. S., Liu T., Thakur A., Sawicki K. T., Prasad S. V. N. et al. (2016). Reduction in mitochondrial iron alleviates cardiac damage during injury. *EMBO Mol. Med.* 8, 247-267. 10.15252/emmm.201505748 26896449 PMC4772952

[JCS247957C6] Conway M. E. (2020). Emerging moonlighting functions of the branched-chain aminotransferase proteins. *Antioxid. Redox Signal.* [Epub] 10.1089/ars.2020.8118 32635740

[JCS247957C7] Conway M. E. and Lee C. (2015). The redox switch that regulates molecular chaperones. *Biomol. Concepts* 6, 269-284. 10.1515/bmc-2015-0015 26352357

[JCS247957C8] Conway M. E., Coles S. J., Islam M. M. and Hutson S. M. (2008). Regulatory control of human cytosolic branched-chain aminotransferase by oxidation and S-glutathionylation and its interactions with redox sensitive neuronal proteins ±. *Biochemistry* 47, 5465-5479. 10.1021/bi800303h 18419134

[JCS247957C9] Di Gioia M., Spreafico R., Springstead J. R., Mendelson M. M., Joehanes R., Levy D. and Zanoni I. (2020). Endogenous oxidized phospholipids reprogram cellular metabolism and boost hyperinflammation. *Nat. Immunol.* 21, 42-53. 10.1038/s41590-019-0539-2 31768073 PMC6923570

[JCS247957C10] Espinosa-Cantú A., Ascencio D., Herrera-Basurto S., Xu J., Roguev A., Krogan N. J. and DeLuna A. (2018). Protein moonlighting revealed by noncatalytic phenotypes of yeast enzymes. *Genetics* 208, 419-431. 10.1534/genetics.117.300377 29127264 PMC5753873

[JCS247957C11] Gardner P. R., Nguyen D. D. and White C. W. (1994). Aconitase is a sensitive and critical target of oxygen poisoning in cultured mammalian cells and in rat lungs. *Proc. Natl. Acad. Sci. USA* 91, 12248-12252. 10.1073/pnas.91.25.12248 7991614 PMC45414

[JCS247957C12] Grajeda-Iglesias C., Rom O., Hamoud S., Volkova N., Hayek T., Abu-Saleh N. and Aviram M. (2018). Leucine supplementation attenuates macrophage foam-cell formation: studies in humans, mice, and cultured macrophages. *Biofactors* 44, 245-262. 10.1002/biof.1415 29399895

[JCS247957C13] Gross T. J., Kremens K., Powers L. S., Brink B., Knutson T., Domann F. E., Philibert R. A., Milhem M. M. and Monick M. M. (2014). Epigenetic silencing of the human NOS2 gene: rethinking the role of nitric oxide in human macrophage inflammatory responses. *J. Immunol.* 192, 2326-2338. 10.4049/jimmunol.1301758 24477906 PMC3943971

[JCS247957C14] Hara K., Yonezawa K., Weng Q.-P., Kozlowski M. T., Belham C. and Avruch J. (1998). Amino acid sufficiency and mTOR regulate p70 S6 kinase and eIF-4E BP1 through a common effector mechanism. *J. Biol. Chem.* 273, 14484-14494. 10.1074/jbc.273.23.14484 9603962

[JCS247957C15] Harris M., El Hindy M., Usmari-Moraes M., Hudd F., Shafei M., Dong M., Hezwani M., Clark P., House M., Forshaw T. et al. (2020). BCAT-induced autophagy regulates Aβ load through an interdependence of redox state and PKC phosphorylation-implications in Alzheimer's disease. *Free Radic. Biol. Med.* 152, 755-766. 10.1016/j.freeradbiomed.2020.01.019 31982508

[JCS247957C16] Hindy M. E. L. and Conway M. E. (2019). Redox-regulated, targeted affinity isolation of NADH-dependent protein interactions with the branched chain aminotransferase proteins. *Methods Mol. Biol.* 1990, 151-163. 10.1007/978-1-4939-9463-2_13 31148070

[JCS247957C17] Jha A. K., Huang S. C.-C., Sergushichev A., Lampropoulou V., Ivanova Y., Loginicheva E., Chmielewski K., Stewart K. M., Ashall J., Everts B. et al. (2015). Network integration of parallel metabolic and transcriptional data reveals metabolic modules that regulate macrophage polarization. *Immunity* 42, 419-430. 10.1016/j.immuni.2015.02.005 25786174

[JCS247957C18] Kang H., Kerloc'h A., Rotival M., Xu X., Zhang Q., D'Souza Z., Kim M., Scholz J. C., Ko J.-H., Srivastava P. K. et al. (2014). Kcnn4 is a regulator of macrophage multinucleation in bone homeostasis and inflammatory disease. *Cell Rep.* 8, 1210-1224. 10.1016/j.celrep.2014.07.032 25131209 PMC4471813

[JCS247957C19] Kelly B. and Pearce E. L. (2020). Amino assets: how amino acids support immunity. *Cell Metab.* 32, 154-175. 10.1016/j.cmet.2020.06.010 32649859

[JCS247957C20] Kingsbury J. M., Sen N. D. and Cardenas M. E. (2015). Branched-chain aminotransferases control TORC1 signaling in Saccharomyces cerevisiae. *PLoS Genet.* 11, e1005714 10.1371/journal.pgen.1005714 26659116 PMC4684349

[JCS247957C21] Kobayashi E. H., Suzuki T., Funayama R., Nagashima T., Hayashi M., Sekine H., Tanaka N., Moriguchi T., Motohashi H., Nakayama K. et al. (2016). Nrf2 suppresses macrophage inflammatory response by blocking proinflammatory cytokine transcription. *Nat. Commun.* 7, 11624 10.1038/ncomms11624 27211851 PMC4879264

[JCS247957C22] Lauterbach M. A., Hanke J. E., Serefidou M., Mangan M. S. J., Kolbe C.-C., Hess T., Rothe M., Kaiser R., Hoss F., Gehlen J. et al. (2019). Toll-like receptor signaling rewires macrophage metabolism and promotes histone acetylation via ATP-citrate lyase. *Immunity* 51, 997-1011.e7. 10.1016/j.immuni.2019.11.009 31851905

[JCS247957C23] Mackay G. M., Zheng L., van den Broek N. J. F. and Gottlieb E. (2015). Analysis of cell metabolism using LC-MS and isotope tracers. *Methods Enzymol.* 561, 171-196. 10.1016/bs.mie.2015.05.016 26358905

[JCS247957C24] Martin S. B., Reiche W. S., Fifelski N. A., Schultz A. J., Stanford S. J., Martin A. A., Nack D. L., Radlwimmer B., Boyer M. P., Ananieva E. A. et al. (2020). Leucine and branched-chain amino acid metabolism contribute to the growth of bone sarcomas by regulating AMPK and mTORC1 signaling. *Biochem. J.* 477, 1579-1599. 10.1042/BCJ20190754 32297642

[JCS247957C25] Martínez-Reyes I. and Chandel N. S. (2020). Mitochondrial TCA cycle metabolites control physiology and disease. *Nat. Commun.* 11, 102 10.1038/s41467-019-13668-3 31900386 PMC6941980

[JCS247957C26] McBrayer S. K., Mayers J. R., DiNatale G. J., Shi D. D., Khanal J., Chakraborty A. A., Sarosiek K. A., Briggs K. J., Robbins A. K., Sewastianik T. et al. (2018). Transaminase inhibition by 2-hydroxyglutarate impairs glutamate biosynthesis and redox homeostasis in glioma. *Cell* 175, 101-116.e25. 10.1016/j.cell.2018.08.038 30220459 PMC6219629

[JCS247957C27] Meiser J., Krämer L., Sapcariu S. C., Battello N., Ghelfi J., D'Herouel A. F., Skupin A. and Hiller K. (2016). Pro-inflammatory macrophages sustain pyruvate oxidation through pyruvate dehydrogenase for the synthesis of itaconate and to enable cytokine expression. *J. Biol. Chem.* 291, 3932-3946. 10.1074/jbc.M115.676817 26679997 PMC4759172

[JCS247957C28] Mills E. L., Kelly B., Logan A., Costa A. S. H., Varma M., Bryant C. E., Tourlomousis P., Däbritz J. H. M., Gottlieb E., Latorre I. et al. (2016). Succinate dehydrogenase supports metabolic repurposing of mitochondria to drive inflammatory macrophages. *Cell* 167, 457-470.e13. 10.1016/j.cell.2016.08.064 27667687 PMC5863951

[JCS247957C29] Mills E. L., Ryan D. G., Prag H. A., Dikovskaya D., Menon D., Zaslona Z., Jedrychowski M. P., Costa A. S. H., Higgins M., Hams E. et al. (2018). Itaconate is an anti-inflammatory metabolite that activates Nrf2 via alkylation of KEAP1. *Nature* 556, 113-117. 10.1038/nature25986 29590092 PMC6047741

[JCS247957C30] Mondanelli G., Bianchi R., Pallotta M. T., Orabona C., Albini E., Iacono A., Belladonna M. L., Vacca C., Fallarino F., Macchiarulo A. et al. (2017). A relay pathway between arginine and tryptophan metabolism confers immunosuppressive properties on dendritic cells. *Immunity* 46, 233-244. 10.1016/j.immuni.2017.01.005 28214225 PMC5337620

[JCS247957C31] Mor D. E., Sohrabi S., Kaletsky R., Keyes W., Tartici A., Kalia V., Miller G. W. and Murphy C. T. (2020). Metformin rescues Parkinson's disease phenotypes caused by hyperactive mitochondria. *Proc. Natl. Acad. Sci. USA* 117, 26438-26447. 10.1073/pnas.2009838117 33024014 PMC7585014

[JCS247957C32] Mori M. and Gotoh T. (2000). Regulation of nitric oxide production by arginine metabolic enzymes. *Biochem. Biophys. Res. Commun.* 275, 715-719. 10.1006/bbrc.2000.3169 10973788

[JCS247957C33] Murphy M. P. and O'Neill L. A. J. (2018). Krebs cycle reimagined: the emerging roles of succinate and itaconate as signal transducers. *Cell* 174, 780-784. 10.1016/j.cell.2018.07.030 30096309

[JCS247957C34] Neinast M., Murashige D. and Arany Z. (2019). Branched chain amino acids. *Annu. Rev. Physiol.* 81, 139-164. 10.1146/annurev-physiol-020518-114455 30485760 PMC6536377

[JCS247957C35] Orino K., Lehman L., Tsuji Y., Ayaki H., Torti S. V. and Torti F. M. (2001). Ferritin and the response to oxidative stress. *Biochem. J.* 357, 241-247. 10.1042/bj3570241 11415455 PMC1221947

[JCS247957C36] Palmieri E. M., Gonzalez-Cotto M., Baseler W. A., Davies L. C., Ghesquière B., Maio N., Rice C. M., Rouault T. A., Cassel T., Higashi R. M. et al. (2020). Nitric oxide orchestrates metabolic rewiring in M1 macrophages by targeting aconitase 2 and pyruvate dehydrogenase. *Nat. Commun.* 11, 698 10.1038/s41467-020-14433-7 32019928 PMC7000728

[JCS247957C37] Papathanassiu A. E., Ko J.-H., Imprialou M., Bagnati M., Srivastava P. K., Vu H. A., Cucchi D., McAdoo S. P., Ananieva E. A., Mauro C. et al. (2017). BCAT1 controls metabolic reprogramming in activated human macrophages and is associated with inflammatory diseases. *Nat. Commun.* 8, 16040 10.1038/ncomms16040 28699638 PMC5510229

[JCS247957C38] Pereira M., Chen T.-D., Buang N., Olona A., Ko J.-H., Prendecki M., Costa A. S. H., Nikitopoulou E., Tronci L., Pusey C. D. et al. (2019). Acute iron deprivation reprograms human macrophage metabolism and reduces inflammation in vivo. *Cell Rep.* 28, 498-511.e5. 10.1016/j.celrep.2019.06.039 31291584 PMC6635384

[JCS247957C39] Pereira M., Ko J.-H., Logan J., Protheroe H., Kim K.-B., Tan A. L. M., Croucher P. I., Park K.-S., Rotival M., Petretto E. et al. (2020). A trans-eQTL network regulates osteoclast multinucleation and bone mass. *eLife* 9, e55549 10.7554/eLife.55549 32553114 PMC7351491

[JCS247957C40] Regan T., Gill A. C., Clohisey S. M., Barnett M. W., Pariante C. M., Harrison N. A., Consortium M. R. C. I., Hume D. A., Bullmore E. T., Freeman T. C. et al. (2018). Effects of anti-inflammatory drugs on the expression of tryptophan-metabolism genes by human macrophages. *J. Leukoc. Biol.* 103, 681-692. 10.1002/JLB.3A0617-261R 29377288 PMC5918594

[JCS247957C41] Rodriguez A. E., Ducker G. S., Billingham L. K., Martinez C. A., Mainolfi N., Suri V., Friedman A., Manfredi M. G., Weinberg S. E., Rabinowitz J. D. et al. (2019). Serine metabolism supports macrophage IL-1β Production. *Cell Metab.* 29, 1003-1011.e4. 10.1016/j.cmet.2019.01.014 30773464 PMC6447453

[JCS247957C42] Saha S., Shalova I. N. and Biswas S. K. (2017). Metabolic regulation of macrophage phenotype and function. *Immunol. Rev.* 280, 102-111. 10.1111/imr.12603 29027220

[JCS247957C43] Sancak Y., Peterson T. R., Shaul Y. D., Lindquist R. A., Thoreen C. C., Bar-Peled L. and Sabatini D. M. (2008). The rag GTPases bind raptor and mediate amino acid signaling to mTORC1. *Science* 320, 1496-1501. 10.1126/science.1157535 18497260 PMC2475333

[JCS247957C100] Seim G. L., Britt E. C., John S. V., Yeo F. J., Johnson A. R., Eisenstein R. S., Pagliarini D. J. and Fan J. (2019). Two-stage metabolic remodelling in macrophages in response to lipopolysaccharide and interferon-γ stimulation. *Nat Metab.* 1, 731-742. 10.1038/s42255-019-0083-2 32259027 PMC7108803

[JCS247957C101] Shang M., Cappellesso F., Amorim R., Serneels J., Virga F., Eelen G., Carobbio S., Rincon M. Y., Maechler P. et al. (2020). Macrophage-derived glutamine boosts satellite cells and muscle regeneration. *Nature* 10.1038/s41586-020-2857-9 PMC7116844 33116312

[JCS247957C44] Silva L. S., Poschet G., Nonnenmacher Y., Becker H. M., Sapcariu S., Gaupel A. C., Schlotter M., Wu Y., Kneisel N., Seiffert M. et al. (2017). Branched-chain ketoacids secreted by glioblastoma cells via MCT1 modulate macrophage phenotype. *EMBO Rep.* 18, 2172-2185. 10.15252/embr.201744154 29066459 PMC5709768

[JCS247957C45] Sivanand S. and Vander Heiden M. G. (2020). Emerging roles for branched-chain amino acid metabolism in cancer. *Cancer Cell* 37, 147-156. 10.1016/j.ccell.2019.12.011 32049045 PMC7082774

[JCS247957C46] Sun K. A., Li Y., Meliton A. Y., Woods P. S., Kimmig L. M., Cetin-Atalay R., Hamanaka R. B. and Mutlu G. M. (2020a). Endogenous itaconate is not required for particulate matter-induced NRF2 expression or inflammatory response. *eLife* 9, e54877 10.7554/eLife.54877 32255424 PMC7185992

[JCS247957C47] Sun X., Xie Z., Hu B., Zhang B., Ma Y., Pan X., Huang H., Wang J., Zhao X., Jie Z. et al. (2020b). The Nrf2 activator RTA-408 attenuates osteoclastogenesis by inhibiting STING dependent NF-κb signaling. *Redox Biol.* 28, 101309 10.1016/j.redox.2019.101309 31487581 PMC6728880

[JCS247957C48] Swain A., Bambouskova M., Kim H., Andhey P. S., Duncan D., Auclair K., Chubukov V., Simons D. M., Roddy T. P., Stewart K. M. et al. (2020). Comparative evaluation of itaconate and its derivatives reveals divergent inflammasome and type I interferon regulation in macrophages. *Nat. Metab.* 2, 594-602. 10.1038/s42255-020-0210-0 32694786 PMC7378276

[JCS247957C49] Tannahill G. M., Curtis A. M., Adamik J., Palsson-McDermott E. M., McGettrick A. F., Goel G., Frezza C., Bernard N. J., Kelly B., Foley N. H. et al. (2013). Succinate is an inflammatory signal that induces IL-1β through HIF-1α. *Nature* 496, 238-242. 10.1038/nature11986 23535595 PMC4031686

[JCS247957C50] van Teijlingen Bakker N. and Pearce E. J. (2020). Cell-intrinsic metabolic regulation of mononuclear phagocyte activation: findings from the tip of the iceberg. *Immunol. Rev.* 295, 54-567. 10.1111/imr.12848 32242952 PMC10911050

[JCS247957C51] Viola A., Munari F., Sanchez-Rodriguez R., Scolaro T. and Castegna A. (2019). The metabolic signature of macrophage responses. *Front. Immunol.* 10, 1462 10.3389/fimmu.2019.01462 31333642 PMC6618143

[JCS247957C52] Wallace C. and Keast D. (1992). Glutamine and macrophage function. *Metabolism* 41, 1016-1020. 10.1016/0026-0495(92)90130-3 1381459

[JCS247957C53] Wang H., Fedorov A. A., Fedorov E. V., Hunt D. M., Rodgers A., Douglas H. L., Garza-Garcia A., Bonanno J. B., Almo S. C., de Carvalho L. P. S. et al. (2019a). An essential bifunctional enzyme in mycobacterium tuberculosis for itaconate dissimilation and leucine catabolism. *Proc. Natl. Acad. Sci. USA* 116, 15907-15913. 10.1073/pnas.1906606116 31320588 PMC6689899

[JCS247957C54] Wang P., Geng J., Gao J., Zhao H., Li J., Shi Y., Yang B., Xiao C., Linghu Y., Sun X. et al. (2019b). Macrophage achieves self-protection against oxidative stress-induced ageing through the Mst-Nrf2 axis. *Nat. Commun.* 10, 755 10.1038/s41467-019-08680-6 30765703 PMC6376064

[JCS247957C55] Weinberg J. B., Misukonis M. A., Shami P. J., Mason S. N., Sauls D. L., Dittman W. A., Wood E. R., Smith G. K., McDonald B., Bachus K. E. et al. (1995). Human mononuclear phagocyte inducible nitric oxide synthase (iNOS): analysis of iNOS mRNA, iNOS protein, biopterin, and nitric oxide production by blood monocytes and peritoneal macrophages. *Blood* 86, 1184-1195. 10.1182/blood.V86.3.1184.1184 7542498

[JCS247957C56] Wuggenig P., Kaya B., Melhem H., Ayata C. K., Swiss IBD Cohort Investigators, Hruz P., Sayan A. E., Tsumura H., Ito M., Roux J. et al. (2020). Loss of the branched-chain amino acid transporter CD98hc alters the development of colonic macrophages in mice. *Commun. Biol.* 3, 130 10.1038/s42003-020-0842-3 32188932 PMC7080761

[JCS247957C57] Yoon B. R., Oh Y.-J., Kang S. W., Lee E. B. and Lee W.-W. (2018). Role of SLC7A5 in metabolic reprogramming of human monocyte/macrophage immune responses. *Front Immunol.* 9, 53 10.3389/fimmu.2018.00053 29422900 PMC5788887

[JCS247957C58] Zhang D., Tang Z., Huang H., Zhou G., Cui C., Weng Y., Liu W., Kim S., Lee S., Perez-Neut M. et al. (2019). Metabolic regulation of gene expression by histone lactylation. *Nature* 574, 575-580. 10.1038/s41586-019-1678-1 31645732 PMC6818755

[JCS247957C59] Zhenyukh O., Civantos E., Ruiz-Ortega M., Sánchez M. S., Vázquez C., Peiró C., Egido J. and Mas S. (2017). High concentration of branched-chain amino acids promotes oxidative stress, inflammation and migration of human peripheral blood mononuclear cells via mTORC1 activation. *Free Radic. Biol. Med.* 104, 165-177. 10.1016/j.freeradbiomed.2017.01.009 28089725

