# Supplementary material for: BCAT1 affects mitochondrial metabolism independently of leucine transamination in activated human macrophages
Source: J Cell Sci. 2020 Nov 27;133(22):jcs247957. doi: 10.1242/jcs.247957 (PMC7116427; doi:10.1242/jcs.247957)
Supplement: Supplementary information [file joces-133-247957-s1.pdf]

Figure S1

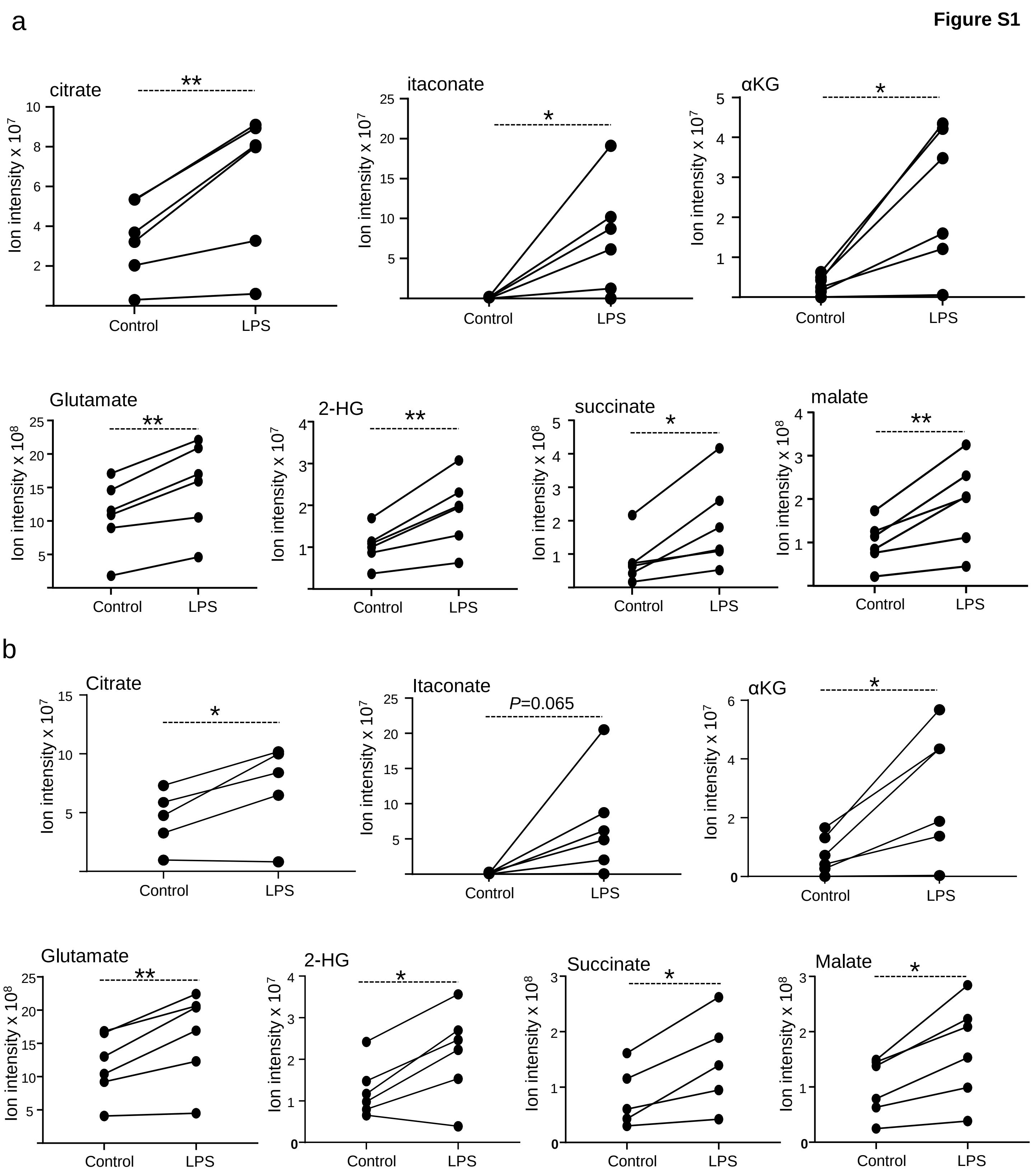

Figure S1. Early LPS activation increases the TCA cycle volume in human macrophages. LC-MS for itaconate, TCA cycle and αKG-derived metabolites in control and LPS (8 hours)-treated hMDMs incubated either with [U-^{13}C]-glucose (**a**) or [U-^{13}C]-glutamine (**b**). Data points are the sum of M+0 and glucose (**a**) or glutamine-derived (**b**) isotopologues; n=6 donors; significance was tested with paired t-test. *, P<0.05; **, P<0.01.

Journal of Cell Science • Supplementary information

Figure S2

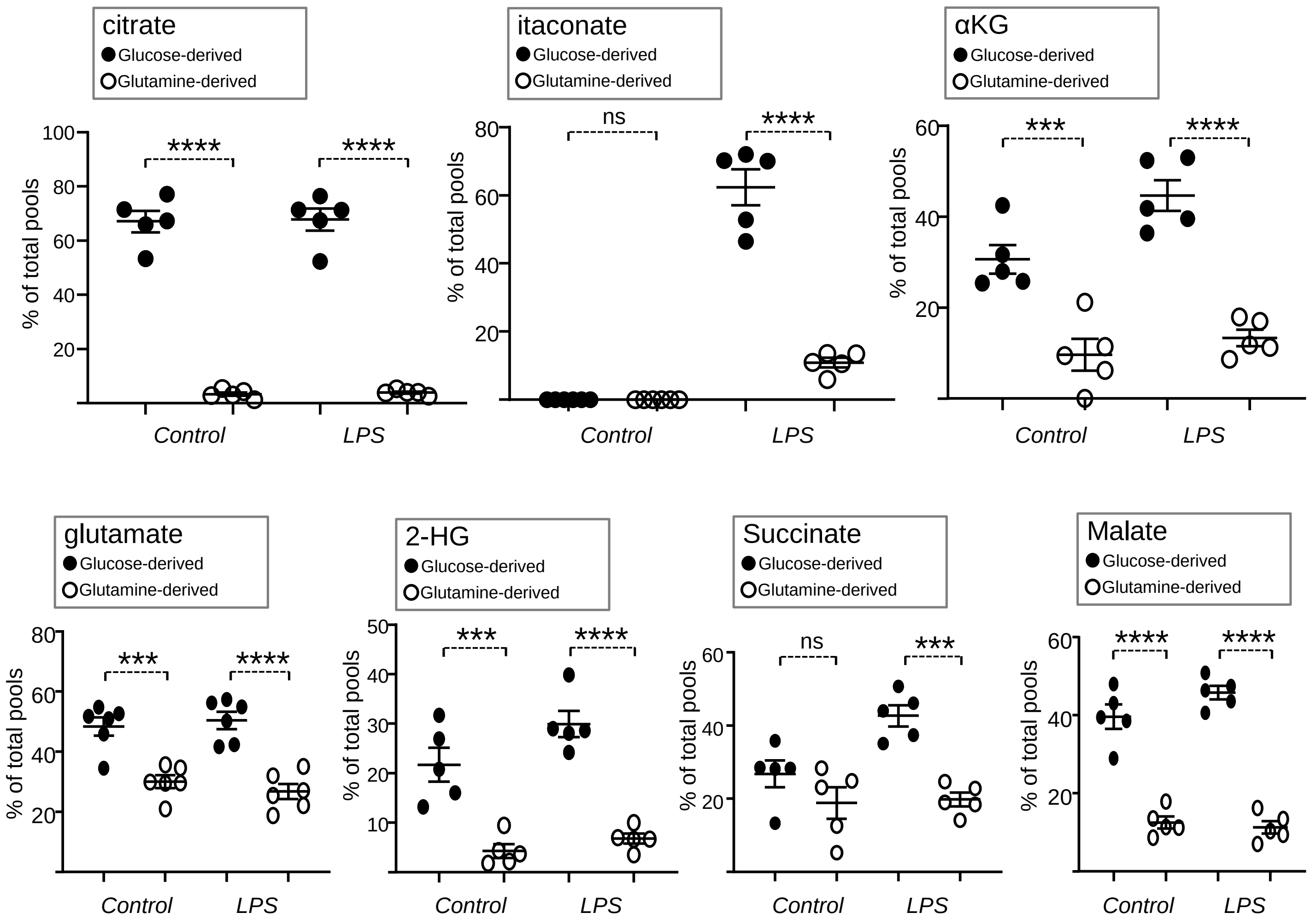

Figure S2. Glucose but not glutamine is a major contributor of the early increase in TCA cycle activity in human macrophages. LC-MS showing the glucose and glutamine-derived isotopologues (as percentage of total pools of metabolites) in control and LPS (8 hours)-treated hMDMs incubated either with [U]-^{13}C-glucose (black circles) or with [U]-^{13}C-glutamine (open circles) ; at least n=5 donors and significance was tested with one-way ANOVA followed by Tukey test; ***, P<0.001; ****, P<0.0001; ns, non-significant.

Journal of Cell Science • Supplementary information

Figure S3

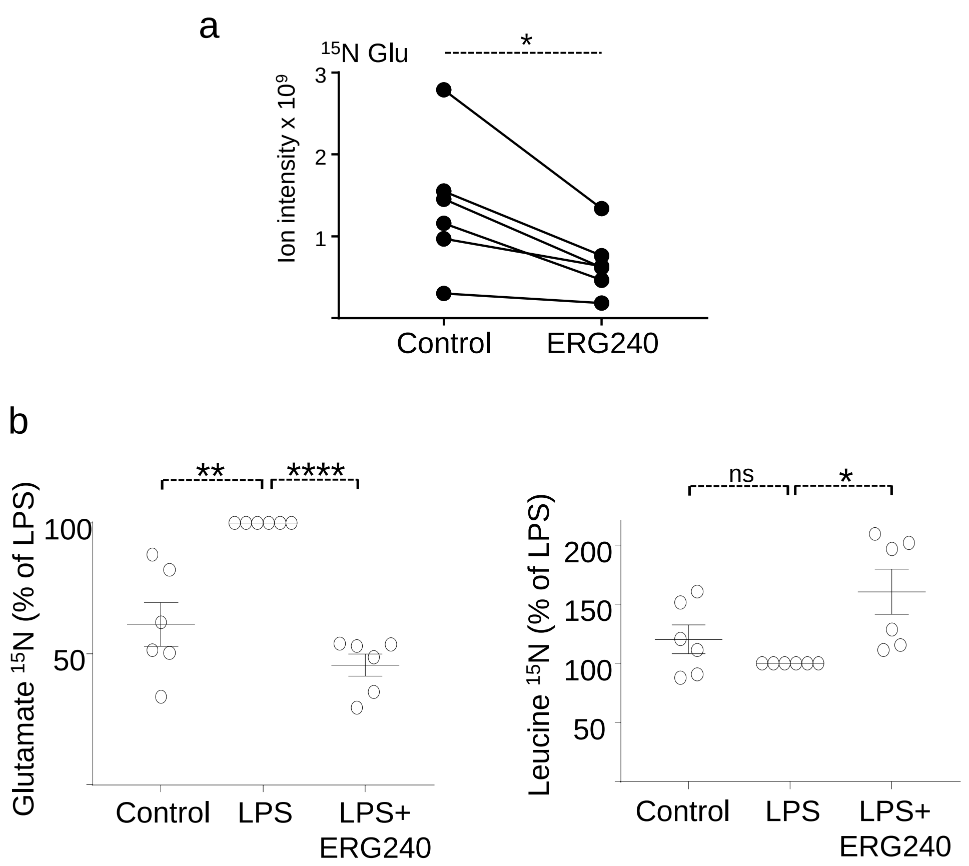

Figure S3. The leucine analogue ERG240 inhibits BCAT1 transamination activity and rescues its LPS-dependent up-regulation. **a**. LC-MS for [^{15}N]-Glutamate in [^{15}N]-Leucine incubated hMDMs in basal and ERG240-treated conditions. **b**. [^{15}N]-Glutamate and $^{[15}$N]-Leucine levels in basal (Control), LPS (8 hours; 100ng/ml) and LPS+ERG240 (8 hours) treatment conditions following incubation with [^{15}N]-Leucine; n=6 donors and significance was tested with paired t-test (**a**) or by one sample t test (**b**); *,P<0.05; **, P<0.01; ****, P<0.0001; ns, non-significant.

Journal of Cell Science • Supplementary information

Figure S4

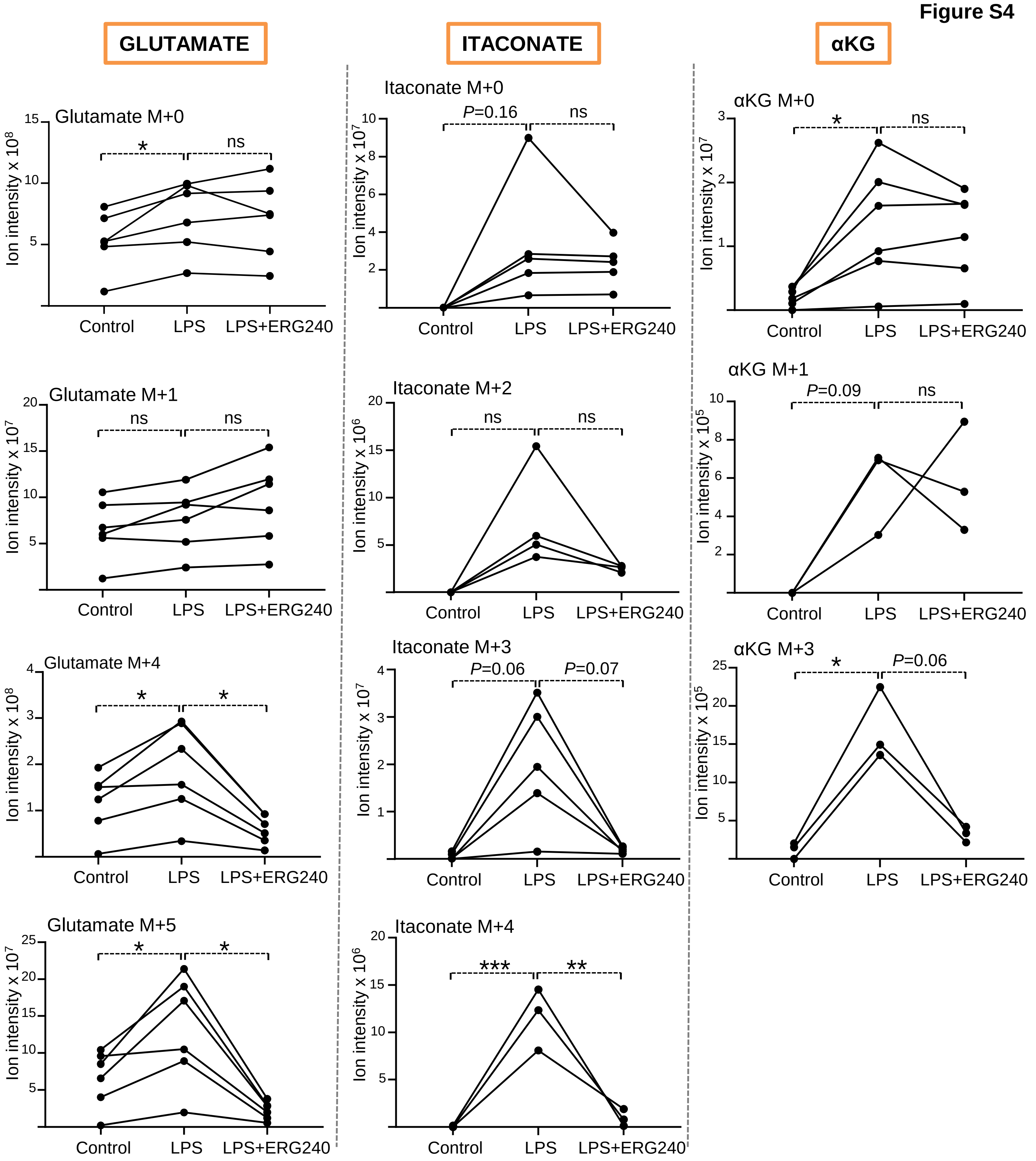

Figure S4. BCAT1 inhibition affects glucose-derived itaconate, αKG and glutamate levels in human macrophages. LC-MS for M+0 and glucose-derived glutamate (left column), itaconate (middle column) and αKG (right column) isotopologues in control (basal), LPS (8 hours) and LPS+ERG240 (8 hours)-treated hMDMs; at least n=3 donors; significance was tested with paired ANOVA followed by Tukey's test. *, P<0.05, **, P<0.01; ***, P<0.001; ns, non-significant.

Journal of Cell Science • Supplementary information

Figure S5

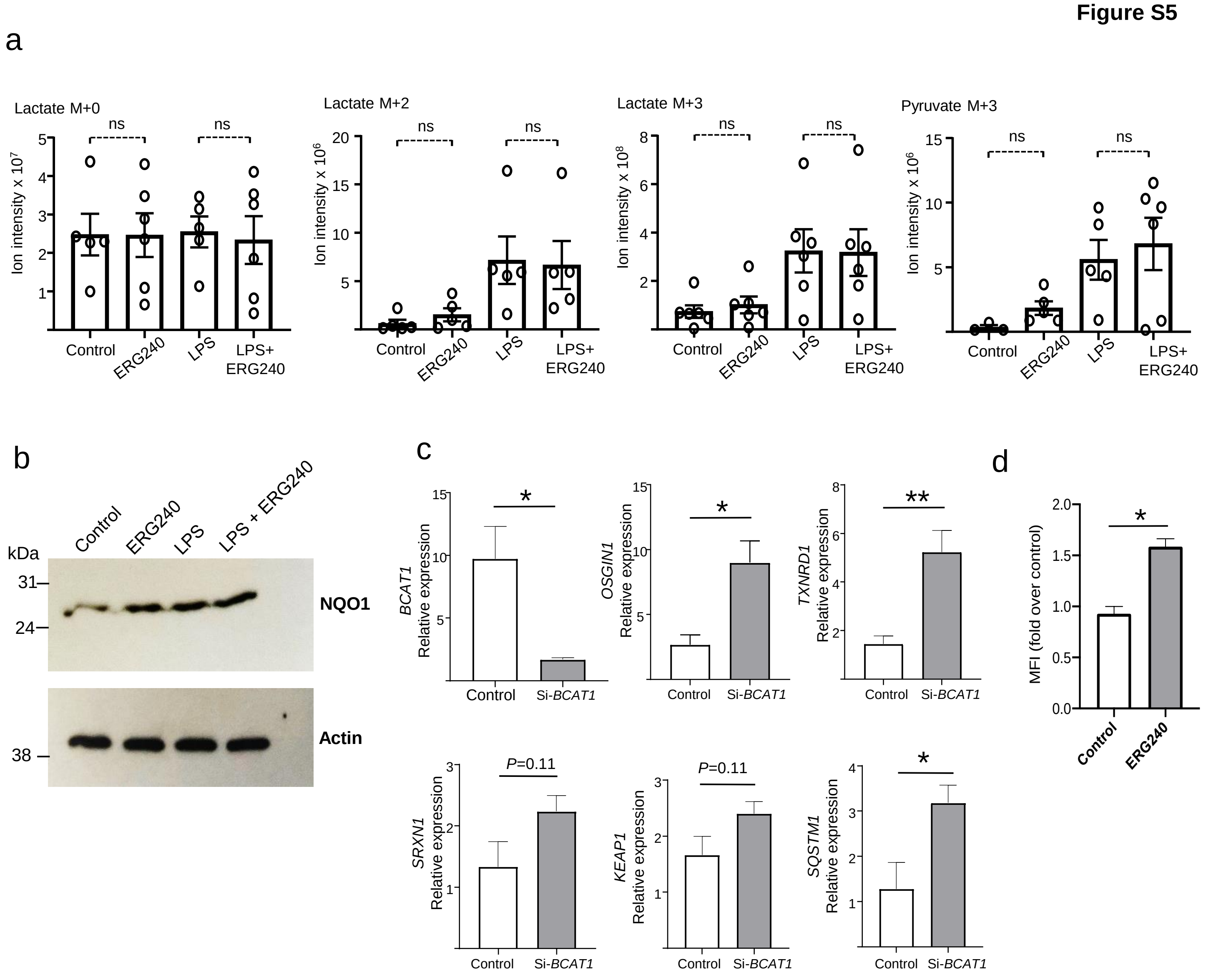

Figure S5. The effect of BCAT1 inhibition on glycolysis and NRF2 pathway in human macrophages. **a**. LC-MS for Lactate M+0, Lactate M+2, Lactate M+3 and Pyruvate M+3 in control (Ctrl), LPS (8h; 100ng/ml), ERG240 and LPS+ERG240-treated hMDMs. **b**. NQO1 Western Blot in control, ERG240, LPS and LPS+ERG240-treated hMDMs. **c**. qRT-PCR for *BCAT1* and NRF2 targets following *BCAT1* siRNA (si-*BCAT1*) or scrambled control (Control). The relative expression levels were normalized to *HPRT* expression levels. **d**. hMDMs were left untreated (Control) or treated 30 min with ERG240. Live cells were analysed by FACS and mean fluorescence intensity (MFI) was quantified as a measure of mitochondrial ROS production. (**a**); at least n=3 donors; significance was tested with one-way ANOVA followed by Tukey's test. (**b**) – (**d**); N=2 donors; significance was tested by t-test. *, $P<0.05$; **, $P<0.01$; ns, non-significant.

Journal of Cell Science • Supplementary information
