# Supplementary material for: BCAT1 affects mitochondrial metabolism independently of leucine transamination in activated human macrophages
Source: J Cell Sci. 2020 Nov 27;133(22):jcs247957. doi: 10.1242/jcs.247957 (PMC7116427; doi:10.1242/jcs.247957)
Supplement: Reviewer comments [file joces-133-247957_review_history.pdf]

BCAT1 affects mitochondrial metabolism independently of leucine transamination in activated human macrophages

Jeong-Hun Ko, Antoni Olona, Adonia E. Papathanassiu, Norzawani Buang, Kwon-Sik Park, Ana S. H. Costa, Claudio Mauro, Christian Frezza and Jacques Behmoaras
DOI: 10.1242/jcs.247957

Editor: Daniel Billadeau

Review timeline

Original submission:	22 April 2020
Editorial decision:	1 July 2020
First revision received:	17 September 2020
Editorial decision:	23 October 2020
Second revision received:	26 October 2020
Accepted:	26 October 2020

Original submission

First decision letter

MS ID#: JOCES/2020/247957

MS TITLE: BCAT1 inhibition elicits oxidative stress and affects mitochondrial metabolism independently of leucine transamination in human macrophages

AUTHORS: Jeong-Hun Ko, Adonia E. Papathanassiu, Norzawani Buang, Kwon-Sik Park, Ana S. H. Costa, Claudio Mauro, Christian Frezza, and Jacques Behmoaras
ARTICLE TYPE: Research Article

We have now reached a decision on the above manuscript.

To see the reviewers' reports and a copy of this decision letter, please go to: https://submit-jcs.biologists.org and click on the 'Manuscripts with Decisions' queue in the Author Area. (Corresponding author only has access to reviews.)

As you will see, the reviewers raise a number of substantial criticisms that prevent me from accepting the paper at this stage. They suggest, however, that a revised version might prove acceptable, if you can address their concerns. If you think that you can deal satisfactorily with the criticisms on revision, I would be pleased to see a revised manuscript. We would then return it to the reviewers.

We are aware that you may currently be unable to access the lab to undertake experimental revisions. If it would be helpful, we encourage you to contact us to discuss your revision in greater detail. Please send us a point-by-point response indicating where you are able to address concerns raised (either experimentally or by changes to the text) and where you will not be able to do so within the normal timeframe of a revision. We will then provide further guidance. Please also note that we are happy to extend revision timeframes as necessary.

Please ensure that you clearly highlight all changes made in the revised manuscript. Please avoid using 'Tracked changes' in Word files as these are lost in PDF conversion.

© 2020. Published by The Company of Biologists under the terms of the Creative Commons Attribution License (https://creativecommons.org/licenses/by/4.0/).

I should be grateful if you would also provide a point-by-point response detailing how you have dealt with the points raised by the reviewers in the 'Response to Reviewers' box. Please attend to all of the reviewers' comments. If you do not agree with any of their criticisms or suggestions please explain clearly why this is so.

Reviewer 1

Advance summary and potential significance to field

In the present work, Ko et al investigate the contribution of branched chain amino acid (BCAA) Leucine to the TCA cycle activation mediated by LPS. Using mass spectrometry, they reveal that glutamine does not act as a source of carbon for the TCA cycle and confirm that LPS activated macrophages rely on glycolysis. Using metabolomics analysis, they reveal that glutamate production is increased after LPS challenge. Based on this observation, they hypothesis that BCAA metabolism is involved in the early stage of metabolic reprograming.
N15 tracing reveals that leucine oxidation is not required but the chemical inhibitor ERG240 demonstrates that BCAT1 regulates the TCA cycle volume. To precise the role of BCAT1 activity during metabolism reprograming, the authors show that ERG240 induces anti-oxidative stress pathways mostly through NRF2. No work is done on anti-inflammatory response. The work is sound but lacks some important controls. I command the authors for utilizing human primary macrophages.
Knowing the carbon source used by macrophages while undergoing LPS-induced metabolic reprograming is certainly of interest for the community. What is more interesting to me is the results presented in Figure 4. If I understand correctly, the authors show that LPS challenge does not stabilized NRF2.
Instead, BCAT1 inhibition is required to trigger an anti-oxidant response. I think that it is generally accepted that LPS stabilizes NRF2, even shortly after challenging macrophages. For instance, Mills and colleague have reported that LPS induces the production of itaconate through IRG1 and that itaconate regulates anti-oxidant program via the alkylation of KEAP1 and the subsequent accumulation of NRF2. The authors have shown in a previous publication that BCAT1 activity controls the amount of IRG1 and itaconate and their model does not suggest a correlation between itaconate level and NRF2 stabilization. This apparent contradiction requires more work to be explained.

Comments for the author

Major comments
-Results from Figure 4 are surprising. Here the authors report that NRF2 accumulation is only seen if BCAT1 activity is inhibited by ERG240. Controlling for species specificity (human MDMs vs mouse BMDMs) and timing seems important to me. I suggest that the authors repeat at least the Western Blot experiments with BMDMs and MDMs challenged with LPS for 6h, 12h and 24h.
Moreover, the model proposed by Mills and colleague suggests that itaconate directly regulates KEAP1 function. This manuscript does not suggest such a connection. Testing the effect of knocking down IRG1 on NRF2 stabilization could help delineate BCAT1 action.
At the very least, the authors should mention the difference and speculated about possible explanation in the Discussion section.
-In Figure 4e: the authors measure the effect of BCAT1 inhibition on ROS production after PMA treatment. The entire manuscript is based on LPS treatment so for the sake of clarity and homogeneity I suggest that the authors repeat this experiment with LPS. It’s an easy experiment therefore matching the number of donors used for the metabolic tracing should not be a problem and will give the opportunity to run statistical analysis.
-the authors claim that the inhibition of BCAT1 activity triggers an anti-oxidant/inflammatory response, but no cytokines are measured. I suggest that the authors co-treat MDMs with LPS and ERG240 and measure commonly induced cytokines (TNF-a, IL-6, type I interferon, IL1-b, IL-10…) at the mRNA and protein level.
-Figure 4f: Could the authors explain why they chose 16h of LPS treatment to measure Ferritine while the rest of the manuscript is done after 8h?

© 2020. Published by The Company of Biologists under the terms of the Creative Commons Attribution License (https://creativecommons.org/licenses/by/4.0/).

-Figure 4g: this experiment lacks LPS alone condition. This experiment was done on two different donors, it's an easy experiment therefore matching the number of donors used for the metabolic tracing should not be a problem and will give the opportunity to run statistical analysis.
-In Figure 3: it would be interesting to see the effect of ERG240 alone versus the control condition.
-Supplementary Figure 4 does not display the control condition. This parameter is important to judge the efficacy of ERG240. The same goes for Figure 4d.

Minor comments
The title of the article is misleading. As I understand it, BCAT1 inhibition elicits an anti-oxidative response.
-In the main text, the authors mention not finding Leucine-derived 13C atoms in the TCA cycle intermediates (Figure 2E). This panel is a schematic representation of their hypothesis. The authors should consider adding these data in a new Figure or in Supplementary Figure.
-The manuscript would benefit from technical clarification. Figure legends related to metabolic tracing could be easier to read if the authors explain what is "stacked glucose-derived isotopologue" and M+0, M+1 etc metabolites.
These modifications would make the manuscript more accessible to the general audience.
-In Figure 3: the authors state "we confirmed the decreased glucose-derived M+1 itaconate that we reported previously". I might be wrong, but I don't see the data.
-the LPS dose used could be mentioned in the manuscript and not only in the Materiel and Methods section.
-The manuscript would benefit from repeating and quantifying the Western Blot.
- I might have not seen them but the complete Western Blot does not appear on the Supplementary Figure. If I am not wrong, please add them.
Figure 4g: It might be a compression/PDF conversion effect but the results from this panel are difficult to read, at least for the line corresponding to mitochondrial aconitase activity. Is there a way to improve the quality of the figure? Is there a way to quantify the intensity of the bands and plot them on a graph that would supplement the gel?
-Some sentences from the discussion are convoluated and difficult to follow.
For e.g. "Thus, loss in aconitase activity has been commonly used as a biomarker of oxidative damageand, in keeping with these findings, we report that pharmacological inhibition of BCAT1 activity is accompanied by by a progressive loss of mitochondrial and cytoplasmic aconitase activity, rescued by co-treatment with LPS and ERG240, presumably because of the induction of antioxidant NRF2 response."

Reviewer 2

Advance summary and potential significance to field

The manuscript by Ko et al. investigate the role of BCAT1 in mitochondrial metabolism in human monocyte-derived macrophages. Using isotope tracing, the authors show that glucose, but not glutamine, was the major fuel source that supports TCA cycle activity during LPS stimulation. Despite an increase in BCAT1 activity, there was no leucine-derived TCA cycle intermediates detected in LPS-stimulated macrophages suggesting the lack of contribution of leucine transamination to mitochondrial metabolism during the cell activation. However, when using the leucine analogue ERG240 to inhibit BCAT1, glucose-derived TCA intermediates including itaconate and alpha-ketoglutarate was reduced. The authors argued this indirect effect is due to the induction of anti-oxidant response that control TCA cycle activity.

While the authorsÂ' findings suggest a moonlight function of BCAT1 in addition to its well-described role in BCAA transamination, the link between the control of mitochondrial metabolism (i.e. TCA cycle) and Nrf2 anti-oxidant response induced by BCAT1 inhibition is poor, and whether macrophage pro-inflammatory polarization is regulated by this anti-oxidant response is not addressed. Also, it would be nice if the authors could provide a bit more mechanistic insight into how BCAT1 inhibition induces Nrf2 anti-oxidant response.

© 2020. Published by The Company of Biologists under the terms of the Creative Commons Attribution License (https://creativecommons.org/licenses/by/4.0/).

Comments for the author

Specific points:
1. In Figure 3a, the authors show that BCAT1 inhibition reduced glucose-derived TCA intermediates. Is this effect specific for TCA cycle or the glucose anaplerotic pathway (i.e. glycolysis)? Did the authors also check the effect of BCAT1 inhibition on glycolysis (e.g. pyruvate, lactate, etc.)?

2. The data on Nrf2 activation by BCAT1 inhibition is rather weak. The authors should also measure other Nrf2 targets such as NQO1 protein level and/or activity. The authors can also consider to use ARE reporter to confirm if BCAT1 inhibition activates Nrf2 at signaling level.

3. It is very unclear whether the effect of BCAT1 inhibition on TCA cycle activity (i.e. reduced intermediates) is mediated via the induction of anti-oxidant response as the authors suggested. Can the authors block Nrf2 activation (e.g. using Nrf2 inhibitor) to see if the effect of BCAT1 inhibition on TCA cycle activity is impaired? Alternatively, would ROS inhibition using anti-oxidants such as NAC have the similar effect on TCA cycle.

4. To tighten the argument of BCAT1 inhibition on macrophage pro-inflammatory polarization, in addition to pathway analysis findings (Figure 4b), more specific data for inflammatory response that is inhibited by BCAT1 inhibition should be included. It would be also interesting to see if Nrf2 anti-oxidant response is required for mediating the anti-inflammatory response.

5. In Figure 4e, the authors show that BCAT1 inhibition reduced PMA-induced ROS. To be also consistent with other experiments, the level of ROS should also be assessed in cells following LPS stimulation.

6. Again it should also include LPS alone condition (i.e. without BCAT1 inhibition) in Figure 4g.

7. The manuscript title states “BCAT1 inhibition elicits oxidative stress and affects…..”. Since the data suggest the opposite (e.g. inhibition of ROS production by ERG240), should it be “elicit oxidative stress response…” or “elicit anti-oxidant response…”?

First revision

Author response to reviewers' comments

- **Reviewer 1 Advance Summary and Potential Significance to Field:**

In the present work, Ko et al investigate the contribution of branched chain amino acid (BCAA) Leucine to the TCA cycle activation mediated by LPS. Using mass spectrometry, they reveal that glutamine does not act as a source of carbon for the TCA cycle and confirm that LPS activated macrophages rely on glycolysis. Using metabolomics analysis, they reveal that glutamate production is increased after LPS challenge. Based on this observation, they hypothesis that BCAA metabolism is involved in the early stage of metabolic reprograming. N15 tracing reveals that leucine oxidation is not required but the chemical inhibitor ERG240 demonstrates that BCAT1 regulates the TCA cycle volume. To precise the role of BCAT1 activity during metabolism reprograming, the authors show that ERG240 induces anti-oxidative stress pathways mostly through NRF2. No work is done on anti-inflammatory response. The work is sound but lacks some important controls. I command the authors for utilizing human primary macrophages. Knowing the carbon source used by macrophages while undergoing LPS-induced metabolic reprograming is certainly of interest for the community. What is more interesting to me is the results presented in Figure 4. If I understand correctly, the authors show that LPS challenge does not stabilized NRF2. Instead, BCAT1 inhibition is required to trigger an anti-oxidant response. I think that it is generally accepted that LPS stabilizes NRF2, even shortly after challenging macrophages. For instance, Mills and colleague have reported that LPS induces the production of itaconate through IRG1 and that itaconate regulates anti-oxidant program via the alkylation of KEAP1 and the subsequent accumulation of NRF2. The authors have shown in a previous publication that BCAT1 activity controls the amount of IRG1 and itaconate and their model

© 2020. Published by The Company of Biologists under the terms of the Creative Commons Attribution License (https://creativecommons.org/licenses/by/4.0/).

does not suggest a correlation between itaconate level and NRF2 stabilization. This apparent contradiction requires more work to be explained.

We thank the reviewer for the careful reading and very constructive feedback on the manuscript, which we believe has strengthened the message overall. We agree with the reviewer that LPS-induced metabolic reprogramming in human macrophages is of interest and the manuscript needs revision on (i) including appropriate controls (ii) clarification of the anti-inflammatory effects of BCAT1 inhibition, (iii) clarification of the link between itaconate and NRF2 in view of our previously published results on BCAT1 activity and IRG1/itaconate.

We have addressed these 3 points in addition to the specific comments outlined below.

Reviewer 1 Comments for the Author:

Major comments

1. Results from Figure 4 are surprising. Here the authors report that NRF2 accumulation is only seen if BCAT1 activity is inhibited by ERG240. Controlling for species specificity (human MDMs vs mouse BMDMs) and timing seems important to me. I suggest that the authors repeat at least the Western Blot experiments with BMDMs and MDMs challenged with LPS for 6h, 12h and 24h. Moreover, the model proposed by Mills and colleague suggests that itaconate directly regulates KEAP1 function. This manuscript does not suggest such a connection. Testing the effect of knocking down IRG1 on NRF2 stabilization could help delineate BCAT1 action. At the very least, the authors should mention the difference and speculated about possible explanation in the Discussion section.

The reviewer is revealing a crucial point that needs addressing in view of the recent findings on the immuno-modulatory role of IRG1/itaconate in macrophages. Indeed, itaconate has been shown to activate the transcription factor NRF2 through alkylation of cysteine residues of KEAP1 during LPS stimulation in mouse BMDMs[1]. NRF2 has been shown to be an anti-inflammatory transcription factor in mouse BMDMs[1,2] In addition, Mills et *al.* incubated the cell permeable itaconate derivative 4-octyl itaconate (4-OI) with LPS-stimulated human PBMCs (but not macrophages) to show that it decreases IL-1ß and TNF protein levels in these cells[1].

Our previous findings showed that BCAT1 inhibition down-regulates IRG1 mRNA/protein and itaconate levels in LPS-stimulated human macrophages, while being broadly anti-inflammatory *in vitro* and *in vivo*[3]. Here we show that BCAT1 inhibition activates NRF2 and the reviewer is correct that our results do not support a possible role of itaconate in the induction of NRF2 following BCAT1 inhibition in LPS-stimulated human macrophages.

Our current hypothesis is that BCAT1 inhibition activates NRF2, independently of its down-regulatory effect on IRG1/itaconate in human macrophages. This is in line with recent reports showing that natural endogenous itaconate behaves differently from electrophilic derivatives such as 4-OI[4,5]. In particular, Swain et al., showed that endogenous itaconate is not a robust NRF2 inducer[5], which further supports that BCAT1-mediated NRF2 induction is independently of itaconate in these cells. Specifically, we observed a rapid mitochondrial oxidative stress and aconitase inhibition generated by BCAT1 inhibition, which we think is the likely cause of the NRF2 response (as shown by others recently[6]) and the down-regulation of metabolites positioned between citrate and succinate through the inhibition of redox-sensitive mitochondrial aconitase activity in the TCA cycle. Our hypothesis also implies that NRF2 induction is upstream itaconate production and preliminary data supports this in human macrophages.

The reviewer is also correct to state that human blood monocyte-derived macrophages and mouse bone marrow-derived macrophages could elicit differences in the LPS-mediated NRF2-responses. As the reviewer states, the specie and length of stimulation could explain the difference in anti-oxidant responses observed in macrophages. For instance, we report that LPS does not elicit strong induction of ROS in human macrophages (see also our response to the next point below).

According to the reviewer’s suggestion, all these points are we now discussed in detail in the revised manuscript as part of the limitations of our study (see highlighted newly added paragraph in the discussion).

© 2020. Published by The Company of Biologists under the terms of the Creative Commons Attribution License (https://creativecommons.org/licenses/by/4.0/).

2. In Figure 4e: the authors measure the effect of BCAT1 inhibition on ROS production after PMA treatment. The entire manuscript is based on LPS treatment so for the sake of clarity and homogeneity I suggest that the authors repeat this experiment with LPS. It's an easy experiment therefore matching the number of donors used for the metabolic tracing should not be a problem and will give the opportunity to run statistical analysis.

According to the reviewer's suggestion, we have measured the LPS-mediated ROS responses in human macrophages. The results (see below) showed that, unlike PMA, LPS does not elicit a significant and robust up-regulation of ROS in primary human macrophages. These results were also replicated by others in healthy human monocytes[7] (See Figure S1A in Bulua et al.[7]) and suggest major differences in oxidative capacity between the widely used mouse BMDMs and human monocytes/macrophages in response to LPS. In order to accurately measure the total oxidative capacity of human macrophages, we have thus decided to rely on PMA-induced ROS and its modulation by BCAT1 activity. We agree with the reviewer that all other experiments use LPS and therefore the rationale behind using PMA over LPS is now included in the revised discussion.

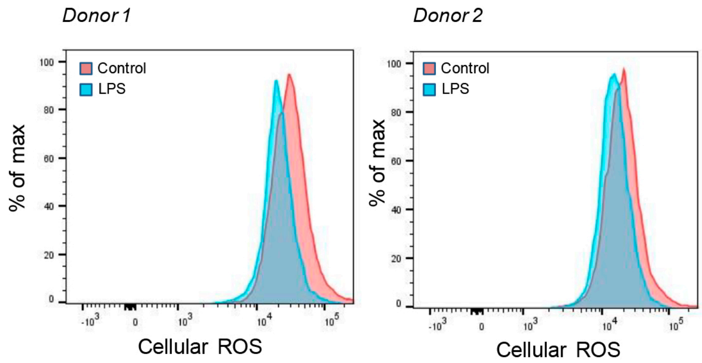

LPS does not elicit robust intracellular ROS in human macrophages. hMDMs from 2 donors were stimulated with LPS (100ng/ml, 8 hours) and cellular ROS was measured using the CellROX Deep Red reagent by FACS. Similar results were found for longer incubation periods with LPS (24h).

3. the authors claim that the inhibition of BCAT1 activity triggers an anti-oxidant/inflammatory response, but no cytokines are measured. I suggest that the authors co-treat MDMs with LPS and ERG240 and measure commonly induced cytokines (TNF-a, IL-6, type I interferon, IL1-b, IL-10…) at the mRNA and protein level.

The reviewer is correct and according to his/her suggestion, we have measured IL-1β protein levels by ELISA in hMDMs co-treated with LPS and ERG240. Furthermore, the overall anti-inflammatory role of BCAT1 inhibition during human macrophage activation (reduction of *TNF* and *NOS2* mRNA; time-dependent reduction in IL-1 β protein levels) was previously described in Papathanassiu et *al.*[3] (Panel B and C below) and the IL-1 β ELISA has confirmed the Western Blot results (see Panels A and B below).

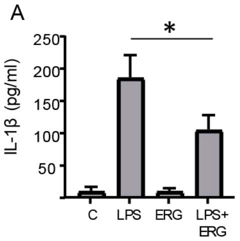

BCAT1 inhibition causes an anti-inflammatory response in LPS-stimulated human macrophages. IL-1b ELISA (**A**) and Western Blot (**B**) analysis in control (untreated), LPS, LPS+ERG240 treated hMDMs. ELISA (this manuscript) was performed 24h following stimulation while the Western Blot (Papathanassiu et al.) was performed at the indicated times following

© 2020. Published by The Company of Biologists under the terms of the Creative Commons Attribution License (https://creativecommons.org/licenses/by/4.0/).

stimulation with LPS and/or ERG240. **B**. Relative expression of *TNF*, *IL6*, *NOS2* and *PTGS2* (normalized to HPRT;) measured by qRT-PCR in control (basal), ERG240-treated, LPS-treated and LPS+ERG240 treated hMDMs. At least, n=3 donor hMDMs were used.

Figure provided for reviewer has been removed. It showed Supplementary Fig. 1b and 1f from Papathanassiu et al. (2017) BCAT1 controls metabolic reprogramming in activated human macrophages and is associated with inflammatory diseases. Nat Commun. 8:16040. (doi: 10.1038/ncomms16040).

We agree with the reviewer that the title of the results describing Figure 4 states '*Inhibition of BCAT1 activity activates NRF2 and triggers anti-oxidant/inflammatory responses*' without showing nor mentioning the results presented below. Hence we have changed the title of the paragraph into '*Inhibition of BCAT1 activity activates NRF2 and triggers anti-oxidant responses*'. This is because the anti-inflammatory effects of BCAT1 inhibition were mostly confirmatory of previously published data (Panel A and Figure 4b in the manuscript) and the novelty of the current paper is NRF2 induction and subsequent anti-oxidant responses upon BCAT1 inhibition (see revised Figure 4 and new Supplementary figure 7 added to further strengthen the NRF2 link).

4. Figure 4f: Could the authors explain why they chose 16h of LPS treatment to measure Ferritine while the rest of the manuscript is done after 8h?

Ferritin heavy chain (H-ferritin) levels were found to be up-regulated in LPS-stimulated human macrophages and, in accordance with others[8], we found that this induction occurs upon 16h of incubation with LPS. As LPS-induced oxidative changes are subtle in human macrophages (see also response to point 2), we used ferritin levels as an additional readout of intracellular oxidative stress as it was previously established[9].

5. Figure 4g: this experiment lacks LPS alone condition. This experiment was done on two different donors, it's an easy experiment therefore matching the number of donors used for the metabolic tracing should not be a problem and will give the opportunity to run statistical analysis.

According to the reviewer's suggestion, we have performed an Aconitase activity gel showing the LPS vs LPS+ERG240 comparison during a time course analysis (see below). However, given the large amounts of protein that this activity gel requires, it was technically challenging to have a quantitative analysis of aconitase activities in 4 conditions (control, ERG240, LPS and LPS+ERG240) during a time course and from 6 donors for robust statistical analysis.

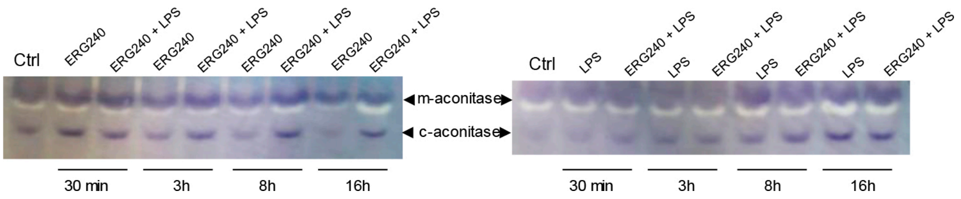

BCAT1 inhibition and ROS-dependent aconitase activities. The left panel shows aconitase activity gel in ERG240-treated (ERG240) and LPS and ERG240 co-treated (LPS+ERG240) hMDMs for the indicated time-points. The right panel shows aconitase activity gel in LPS-treated (LPS) and LPS and ERG240 co-treated (LPS+ERG240) hMDMs for the indicated time- points. m-aconitase, mitochondrial; c-aconitase, cytosolic. The results are representative of two independent experiments from 2 separate donors.

As a result, we have withdrawn Figure 4g from the revised manuscript as it is redundant in highlighting the anti-oxidant role of BCAT1 inhibition, measured by (i) the overall increase of metabolites implicated in the GSH pathway (revised Figure 4d), (ii) reduction of PMA-induced oxidative stress (Figure 4e), (iii) the reduction of H-Ferritin levels (Figure 4f) in activated macrophages.

6. In Figure 3: it would be interesting to see the effect of ERG240 alone versus the control condition.

© 2020. Published by The Company of Biologists under the terms of the Creative Commons Attribution License (https://creativecommons.org/licenses/by/4.0/).

According to the reviewer's suggestion, we have now included the ERG240 alone condition and changed the Figure 3 accordingly. The revised Figure 3 has now been added to the manuscript and the legend modified accordingly.

7. Supplementary Figure 4 does not display the control condition. This parameter is important to judge the efficacy of ERG240. The same goes for Figure 4d.

According to the reviewer's suggestion, we have now included the control (basal) condition in Supplementary Figure 4 and Figure 4d. These revised figures have now been included in the manuscript. Supplementary Figure 4 has been numbered as Supplementary Figure 5 in the revised manuscript.

Minor comments

1. The title of the article is misleading. As I understand it, BCAT1 inhibition elicits an anti-oxidative response.

According to the reviewer's suggestion, we have simplified the main message by changing the title into '*BCAT1 affects mitochondrial metabolism independently of leucine transamination in activated human macrophages*'

2. In the main text, the authors mention not finding Leucine-derived 13C atoms in the TCA cycle intermediates (Figure 2E). This panel is a schematic representation of their hypothesis. The authors should consider adding these data in a new Figure or in Supplementary Figure.

This is a valid point and according to the reviewer's point, we have added the LC-MS data on TCA cycle intermediates in labelled leucine experiment as the Supplementary Figure 3 in the revised manuscript.

3. The manuscript would benefit from technical clarification. Figure legends related to metabolic tracing could be easier to read if the authors explain what is "stacked glucose-derived isotopologue" and M+0, M+1 etc metabolites. These modifications would make the manuscript more accessible to the general audience.

We agree with the reviewer and according to his/her suggestion, these technical sentences were changed into 'Glucose-derived and M+0 metabolites' and 'Glutamine-derived and M+0 metabolites'.

4. In Figure 3: the authors state "we confirmed the decreased glucose-derived M+1 itaconate that we reported previously". I might be wrong, but I don't see the data.

The statement refers to the finding reported in Papathanassiu et al., 2017, showing that BCAT1 inhibition resulted in decreased itaconate levels. The reviewer is correct that the sentence could me misleading as the original publication shows the total itaconate levels and not the glucose-derived isotopologue. The sentence is now amended in the revised version of the manuscript.

5. the LPS dose used could be mentioned in the manuscript and not only in the Materiel and Methods section.

According to the reviewer's suggestion, we have now added the concentration of LPS (100 ng/ml into the figure legends).

6. The manuscript would benefit from repeating and quantifying the Western Blot.

As the NRF2 Western Blot shows a clear effect of BCAT1 inhibition (Figure 4b) which is corroborated by RNA-seq (Figure 4a,b and c), LC-MS data on the GSH pathway metabolites (Figure 4d), new data on BCAT1 siRNA and NQO1 Western Blotting (Supplementary Figure 7), and the anti-oxidant role of ERG240+LPS (Figure 4e and f); we are confident that NRF2 pathway is induced upon BCAT1 inhibition to elicit an anti-oxidant response activated human macrophages. Hence the NRF2 Western Blot is shown as supportive data all assays presented in Figure 4 and Supplementary Figure 7.

© 2020. Published by The Company of Biologists under the terms of the Creative Commons Attribution License (https://creativecommons.org/licenses/by/4.0/).

7. I might have not seen them but the complete Western Blot does not appear on the Supplementary Figure. If I am not wrong, please add them.

We will supply the uncropped Western Blots according the JCS recommendations.

8. Figure 4g: It might be a compression/PDF conversion effect but the results from this panel are difficult to read, at least for the line corresponding to mitochondrial aconitase activity. Is there a way to improve the quality of the figure? Is there a way to quantify the intensity of the bands and plot them on a graph that would supplement the gel?

Figure 4g was withdrawn from the revised manuscript (see also Response to Major point 5.) as it is redundant in highlighting the anti-oxidant role of BCAT1 inhibition, measured by (i) the overall increase of metabolites implicated in the GSH pathway (Figure 4d), (ii) reduction of PMA-induced oxidative stress (Figure 4e), (iii) the reduction of H-Ferritin levels (Figure 4f) in activated macrophages.

Some sentences from the discussion are convoluated and difficult to follow. For e.g. "Thus, loss in aconitase activity has been commonly used as a biomarker of oxidative damage and, in keeping with these findings, we report that pharmacological inhibition of BCAT1 activity is accompanied by by a progressive loss of mitochondrial and cytoplasmic aconitase activity, rescued by co-treatment with LPS and ERG240, presumably because of the induction of antioxidant NRF2 response."

The sentence has now been deleted from the revised manuscript.

- **Reviewer 2 Advance Summary and Potential Significance to Field:**

The manuscript by Ko et al. investigate the role of BCAT1 in mitochondrial metabolism in human monocyte-derived macrophages. Using isotope tracing, the authors show that glucose, but not glutamine, was the major fuel source that supports TCA cycle activity during LPS stimulation. Despite an increase in BCAT1 activity, there was no leucine-derived TCA cycle intermediates detected in LPS-stimulated macrophages, suggesting the lack of contribution of leucine transamination to mitochondrial metabolism during the cell activation. However, when using the leucine analogue ERG240 to inhibit BCAT1, glucose-derived TCA intermediates including itaconate and alpha-ketoglutarate was reduced. The authors argued this indirect effect is due to the induction of anti-oxidant response that control TCA cycle activity.

While the authors' findings suggest a moonlight function of BCAT1 in addition to its well-described role in BCAA transamination, the link between the control of mitochondrial metabolism (i.e. TCA cycle) and Nrf2 anti-oxidant response induced by BCAT1 inhibition is poor, and whether macrophage pro-inflammatory polarization is regulated by this anti-oxidant response is not addressed. Also, it would be nice if the authors could provide a bit more mechanistic insight into how BCAT1 inhibition induces Nrf2 anti-oxidant response.

We thank the reviewer for the accurate summary of our findings and the constructive review. We also thank the reviewer for experimental suggestions that consolidated the findings (the BCAT1-TCA cycle and BCAT1-NRF2 links). We revised the manuscript to not to claim direct links between the BCAT1-mediated Nrf2 anti-oxidant responses and (i) control of TCA cycle, (ii) modulatory effects on macrophage polarization. Addressing both points fully would necessitate the elucidation of immunomodulatory and metabolic effects of NRF2-driven antioxidant responses in human macrophages, which is a significant new undertaking. We have therefore acknowledged the limitations of our study and provided a very detailed discussion about one possible mechanism through which BCAT1 inhibition could control the TCA cycle activity and macrophage polarization. The reviewer's all other points were addressed, and the manuscript revised accordingly.

© 2020. Published by The Company of Biologists under the terms of the Creative Commons Attribution License (https://creativecommons.org/licenses/by/4.0/).

Reviewer 2 Comments for the Author:

<u>Specific points:</u>

1. In Figure 3a, the authors show that BCAT1 inhibition reduced glucose-derived TCA intermediates. Is this effect specific for TCA cycle or the glucose anaplerotic pathway (i.e. glycolysis)? Did the authors also check the effect of BCAT1 inhibition on glycolysis (e.g. pyruvate, lactate, etc.)?

According to the reviewer's recommendation, we have measured the effect of BCAT1 inhibition on glucose-derived pyruvate and lactate levels (see below and Supplementary Figure 6 in the revised manuscript). The results have shown that the glycolysis is not affected at 8 hours following LPS stimulation, while some glucose-derived TCA cycle metabolites are significantly down-regulated (Figure 3). These new results have been added into the revised manuscript as Supplementary Figure 6 and. We thank the reviewer for his/her suggestion, which helped highlighting the specificity of the BCAT1 inhibition with regards to two major metabolic pathways in human macrophages.

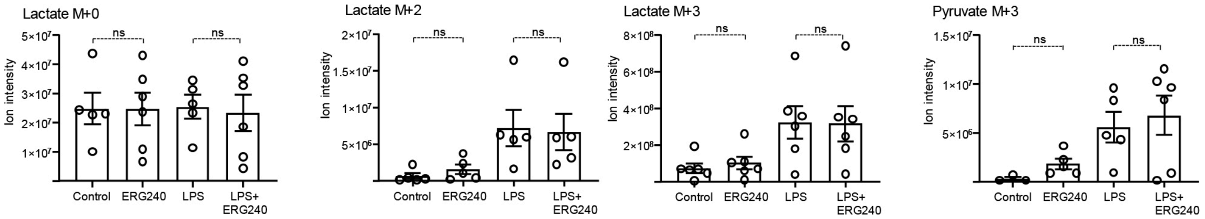

Supplementary Figure 6. BCAT1 inhibition does not affect glucose-derived pyruvate and lactate levels in activated human macrophages. LC-MS for Lactate M+0, Lactate M+2, Lactate M+3 and Pyruvate M+3 in control (Ctrl), LPS (8h; 100ng/ml), ERG240 and LPS+ERG240-treated hMDMs; at least n=3 donors; significance was tested with one-way ANOVA followed by Tukey's test. ns, non-significant.

2. The data on Nrf2 activation by BCAT1 inhibition is rather weak. The authors should also measure other Nrf2 targets such as NQO1 protein level and/or activity. The authors can also consider to use ARE reporter to confirm if BCAT1 inhibition activates Nrf2 at signaling level.

According to the reviewer's suggestion, we measured NQO1 protein levels following BCAT1 inhibition in hMDMs. To further strengthen the link, we have tested a non-pharmacological way of interfering with BCAT1 activity. We thus performed transient silencing of *BCAT1* in hMDMs and measured the expression level of genes associated with *NRF2* pathway (*OSGIN1*, *TXNRD1*, *SRXN1*, *KEAP1*, *SQSTM1*) by qRT-PCR. The results showed that BCAT1 inhibition, even in the absence of LPS stimulation, activates the NRF2 pathway genes (see below and Supplementary Figure 7), confirming the results presented in Figure 4a and b.

The link between BCAT1 activity and NRF2 is now shown by (i) NRF2 Western Blot (Figure 4b) (ii) RNA- seq of NRF2 targets (Figure 4a,b and c), (iii) LC-MS data of GSH pathway (Figure 4d), (iv) NQO1 Western Blot and NRF2 target gene up-regulation upon *BCAT1* silencing (Supplementary Figure 7). Taken together, these data show thats BCAT1 inhibition activates NRF2 and is anti-oxidant (Figure 4e and f) in activated human macrophages.

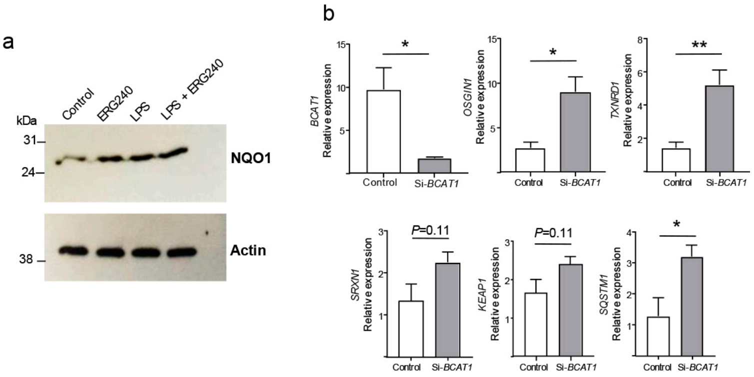

© 2020. Published by The Company of Biologists under the terms of the Creative Commons Attribution License (https://creativecommons.org/licenses/by/4.0/).

Supplementary Figure 7. BCAT1 protein/activity levels are upstream NRF2 pathway. **a**. NQO1 Western Blot in control, ERG240, LPS and LPS+ERG240-treated hMDMs **b**. qRT-PCR for *BCAT1* and NRF2 targets following *BCAT1* siRNA (si-*BCAT1*) or scrambled control (Control). The relative expression levels were normalized to HPRT expression levels. N=2 donors; significance was tested by t-test. *, P<0.05; **, P<0.01.

3. It is very unclear whether the effect of BCAT1 inhibition on TCA cycle activity (i.e. reduced intermediates) is mediated via the induction of anti-oxidant response as the authors suggested. Can the authors block Nrf2 activation (e.g. using Nrf2 inhibitor) to see if the effect of BCAT1 inhibition on TCA cycle activity is impaired? Alternatively, would ROS inhibition using anti-oxidants such as NAC have the similar effect on TCA cycle.

We apologise for the misleading suggestions stating that the reduction in TCA cycle activity upon BCAT1 inhibition is due to NRF2-mediated antioxidant responses. We went through the manuscript and corrected the text, to not to claim causality between BCAT1-mediated NRF2 induction and the TCA cycle activity (see highlighted text in the abstract).

The mechanisms through which the NRF2-mediated anti-oxidant response are linked to the TCA cycle activity upon BCAT1 inhibition, warrant further investigation. One plausible mechanistic pathway is based on the fact that BCAT1 inhibition induced mitochondrial ROS rapidly (within 30 minutes) in human macrophages (data not shown). Mitochondrial ROS has been recently shown to activate NRF2 in macrophages[6] and could explain the reduction of the TCA cycle activity through inhibition of mitochondrial aconitase (ACO2) activity, which is tightly dependent on ROS levels. This hypothesis is discussed in the revised manuscript (see highlighted text) by stating clearly the limitation of our study (understanding the link between NRF2 and TCA cycle activity).

4. To tighten the argument of BCAT1 inhibition on macrophage pro-inflammatory polarization, in addition to pathway analysis findings (Figure 4b), more specific data for inflammatory response that is inhibited by BCAT1 inhibition should be included. It would be also interesting to see if Nrf2 anti-oxidant response is required for mediating the anti-inflammatory response.

We thank the reviewer for these suggestions, and we have taken them into account. First, the inhibition of pro-inflammatory polarization by BCAT1 was confirmed by measuring IL-1ß levels by ELISA (Panel A below). These results confirmed our previous ones published in Papathanassiu et al[3]. Because these results were confirmatory of the previously published results, here we highlight the effect of BCAT1 inhibition on NRF2 activation and anti-oxidant responses (Figure 4). We have therefore changed the title of the paragraph describing the results of Figure 4 and the edited title reflects accurately the results presented in the revised manuscript.

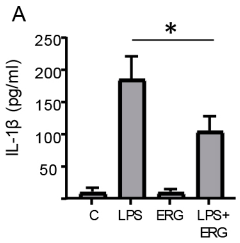

BCAT1 inhibition causes an anti-inflammatory response in LPS-stimulated human macrophages. IL-1b ELISA (**A**) and Western Blot (**B**) analysis in control (untreated), LPS, LPS+ERG240 treated hMDMs. ELISA (this manuscript) was performed 24h following stimulation while the Western Blot (Papathanassiu et al.) was performed at the indicated times following stimulation with LPS and/or ERG240. **B**. Relative expression of *TNF*, *IL6*, *NOS2* and *PTGS2* (normalized to HPRT;) measured by qRT-PCR in control (basal), ERG240-treated, LPS-treated and LPS+ERG240 treated hMDMs. At least, n=3 donor hMDMs were used.

Figure provided for reviewer has been removed. It showed Supplementary Fig. 1b and 1f from Papathanassiu et al. (2017) BCAT1 controls metabolic reprogramming in activated

© 2020. Published by The Company of Biologists under the terms of the Creative Commons Attribution License (https://creativecommons.org/licenses/by/4.0/).

human macrophages and is associated with inflammatory diseases. Nat Commun. 8:16040. (doi: 10.1038/ncomms16040).

Second, we have followed the reviewer advice and attempted an experiment to see whether a chemical NRF2 inhibitor (ML385) can rescue the anti-inflammatory effect of BCAT1 inhibition. Strikingly, we found that, the basal (untreated) macrophages showed a significant induction of IL-1β protein levels and *IRG1* mRNA levels upon dual inhibition of BCAT1 and NRF2 (see below – Panel A and B). However, NRF2 inhibition did not rescue IL-1β levels in (LPS+ERG240) - stimulated macrophages (Panel A). These results suggest that BCAT1 inhibition loses its anti-inflammatory effect if NRF2 is not engaged downstream. These preliminary results necessitate the identification of the exact immunomodulatory effects of NRF2 in human macrophages and their study in the context of BCAT1 inhibition (see revised discussion on the limitations of our study).

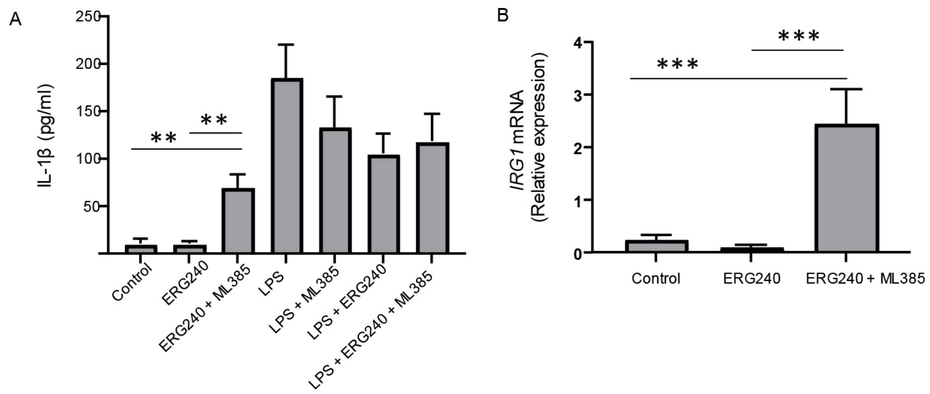

The role of NRF2 in BCAT1 inhibition. IRG1 mRNA (A) and IL-1β ELISA (B) measured following incubation with the different compounds (as indicated; 24h incubation) in hMDMs. ML385 was used at 20 μM; n=3 donor hMDMs were used.

5. In Figure 4e, the authors show that BCAT1 inhibition reduced PMA-induced ROS. To be also consistent with other experiments, the level of ROS should also be assessed in cells following LPS stimulation.

According to the reviewer's suggestion, we have measured the LPS-mediated ROS responses in human macrophages. The results (see below) showed that, unlike PMA, LPS does not elicit a significant and robust up-regulation of ROS in primary human macrophages. These results were also replicated by others in healthy human monocytes[7] (See Figure S1A in Bulua et al.[7]) and suggest major differences in oxidative capacity between the widely used mouse BMDMs and human monocytes/macrophages in response to LPS. In order to accurately measure the total oxidative capacity of human macrophages, we have thus decided to rely on PMA-induced ROS and its modulation by BCAT1 activity. We agree with the reviewer that all other experiments use LPS and therefore the rationale behind using PMA over LPS is now included in the revised discussion.

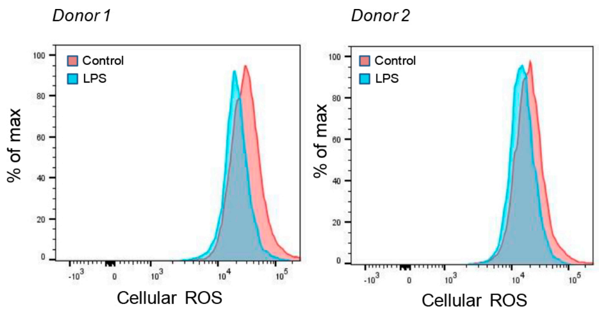

LPS does not elicit robust intracellular ROS in human macrophages. hMDMs from 2 donors were stimulated with LPS (100ng/ml, 8 hours) and cellular ROS was measured using the CellROX Deep Red reagent by FACS. Similar results were found for longer incubation periods with LPS (24h).

© 2020. Published by The Company of Biologists under the terms of the Creative Commons Attribution License (https://creativecommons.org/licenses/by/4.0/).

6. Again it should also include LPS alone condition (i.e. without BCAT1 inhibition) in Figure 4g.

According to the reviewer's suggestion, we have performed an Aconitase activity gel showing the LPS vs LPS+ERG240 comparison during a time course analysis (see below). However, given the large amounts of protein that this activity gel requires, it was technically challenging to have a quantitative analysis of aconitase activities in 4 conditions (control, ERG240, LPS and LPS+ERG240) during a time course and from 6 donors for robust statistical analysis.

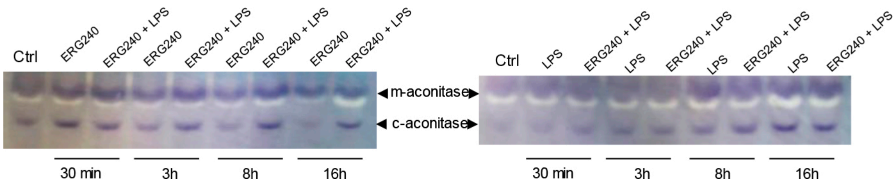

BCAT1 inhibition and ROS-dependent aconitase activities. The left panel shows aconitase activity gel in ERG240-treated (ERG240) and LPS and ERG240 co-treated (LPS+ERG240) hMDMs for the indicated time-points. The right panel shows aconitase activity gel in LPS-treated (LPS) and LPS and ERG240 co-treated (LPS+ERG240) hMDMs for the indicated time- points. m-aconitase, mitochondrial; c-aconitase, cytosolic. The results are representative of two independent experiments from 2 separate donors.

As a result, we have withdrawn Figure 4g from the revised manuscript as it is redundant in highlighting the anti-oxidant role of BCAT1 inhibition, measured by (i) the overall increase of metabolites implicated in the GSH pathway (revised Figure 4d), (ii) reduction of PMA-induced oxidative stress (Figure 4e), (iii) the reduction of LPS-induced H-Ferritin levels (Figure 4f) in activated macrophages.

7. The manuscript title states "BCAT1 inhibition elicits oxidative stress and affects…..". Since the data suggest the opposite (e.g. inhibition of ROS production by ERG240), should it be "elicit oxidative stress response…" or "elicit anti-oxidant response…"?

The reviewer is correct, and the title has now been changed into '*BCAT1 affects mitochondrial metabolism independently of leucine transamination in activated human macrophages*'.

References

1. Mills, E.L., Ryan, D.G., Prag, H.A., Dikovskaya, D., Menon, D., Zaslona, Z., Jedrychowski, M.P., Costa, A.S.H., Higgins, M., Hams, E., Szpyt, J., Runtsch, M.C., King, M.S., McGouran, J.F., Fischer, R., Kessler, B.M., McGettrick, A.F., Hughes, M.M., Carroll, R.G., Booty, L.M., Knatko, E.V., Meakin, P.J., Ashford, M.L.J., Modis, L.K., Brunori, G., Sevin, D.C., Fallon, P.G., Caldwell, S.T., Kunji, E.R.S., Chouchani, E.T., Frezza, C., Dinkova-Kostova, A.T., Hartley, R.C., Murphy, M.P. & O'Neill, L.A. Itaconate is an anti-inflammatory metabolite that activates Nrf2 via alkylation of KEAP1. *Nature* 556: 113-117, 2018.
2. Kobayashi, E.H., Suzuki, T., Funayama, R., Nagashima, T., Hayashi, M., Sekine, H., Tanaka, N., Moriguchi, T., Motohashi, H., Nakayama, K. & Yamamoto, M. Nrf2 suppresses macrophage inflammatory response by blocking proinflammatory cytokine transcription. *Nature communications* 7: 11624, 2016.
3. Papathanassiu, A.E., Ko, J.H., Imprialou, M., Bagnati, M., Srivastava, P.K., Vu, H.A., Cucchi, D., McAdoo, S.P., Ananieva, E.A., Mauro, C. & Behmoaras, J. BCAT1 controls metabolic reprogramming in activated human macrophages and is associated with inflammatory diseases. *Nature communications* 8: 16040, 2017.
4. Sun, K.A., Li, Y., Meliton, A.Y., Woods, P.S., Kimmig, L.M., Cetin-Atalay, R., Hamanaka, R.B. & Mutlu,
G.M. Endogenous itaconate is not required for particulate matter-induced NRF2 expression or inflammatory response. *Elife* 92020.
5. Swain, A., Bambouskova, M., Kim, H., Andhey, P.S., Duncan, D., Auclair, K., Chubukov, V., Simons, D.M., Roddy, T.P., Stewart, K.M. & Artyomov, M.N. Comparative evaluation of itaconate

© 2020. Published by The Company of Biologists under the terms of the Creative Commons Attribution License (https://creativecommons.org/licenses/by/4.0/).

and its derivatives reveals divergent inflammasome and type I interferon regulation in macrophages. *Nat Metab* 2: 594-602, 2020.
6. Wang, P., Geng, J., Gao, J., Zhao, H., Li, J., Shi, Y., Yang, B., Xiao, C., Linghu, Y., Sun, X., Chen, X., Hong, L., Qin, F., Li, X., Yu, J.S., You, H., Yuan, Z., Zhou, D., Johnson, R.L. & Chen, L. Macrophage achieves self-protection against oxidative stress-induced ageing through the Mst-Nrf2 axis. *Nature communications* 10: 755, 2019.
7. Bulua, A.C., Simon, A., Maddipati, R., Pelletier, M., Park, H., Kim, K.Y., Sack, M.N., Kastner, D.L. & Siegel, R.M. Mitochondrial reactive oxygen species promote production of proinflammatory cytokines and are elevated in TNFR1-associated periodic syndrome (TRAPS). *J Exp Med* 208: 519-533, 2011.
8. Recalcati, S., Locati, M., Marini, A., Santambrogio, P., Zaninotto, F., De Pizzol, M., Zammataro, L., Girelli, D. & Cairo, G. Differential regulation of iron homeostasis during human macrophage polarized activation. *Eur J Immunol* 40: 824-835, 2010.
9. Orino, K., Lehman, L., Tsuji, Y., Ayaki, H., Torti, S.V. & Torti, F.M. Ferritin and the response to oxidative stress. *Biochem J* 357: 241-247, 2001.

Second decision letter

MS ID#: JOCES/2020/247957

MS TITLE: BCAT1 affects mitochondrial metabolism independently of leucine transamination in activated human macrophages

AUTHORS: Jeong-Hun Ko, Antoni Olona, Adonia E. Papathanassiu, Norzawani Buang, Kwon-Sik Park, Ana S. H. Costa, Claudio Mauro, Christian Frezza, and Jacques Behmoaras
ARTICLE TYPE: Research Article

We have now reached a decision on the above manuscript.

To see the reviewers' reports and a copy of this decision letter, please go to: https://submit-jcs.biologists.org and click on the 'Manuscripts with Decisions' queue in the Author Area. (Corresponding author only has access to reviews.)

Could you please respond to the requests of reviewer 2. I hope that you will be able to carry these out, because I would like to be able to accept your paper.

We are aware that you may be experiencing disruption to the normal running of your lab that makes experimental revisions challenging.If it would be helpful, we encourage you to contact us to discuss your revision in greater detail. Please send us a point-by-point response indicating where you are able to address concerns raised (either experimentally or by changes to the text) and where you will not be able to do so within the normal timeframe of a revision. We will then provide further guidance. Please also note that we are happy to extend revision timeframes as necessary.

Please ensure that you clearly highlight all changes made in the revised manuscript. Please avoid using 'Tracked changes' in Word files as these are lost in PDF conversion.

I should be grateful if you would also provide a point-by-point response detailing how you have dealt with the points raised by the reviewers in the 'Response to Reviewers' box. Please attend to all of the reviewers' comments. If you do not agree with any of their criticisms or suggestions please explain clearly why this is so.

© 2020. Published by The Company of Biologists under the terms of the Creative Commons Attribution License (https://creativecommons.org/licenses/by/4.0/).

Reviewer 1

Advance summary and potential significance to field

In the present work, Ko et al investigate the contribution of branched chain amino acid (BCAA) Leucine to the TCA cycle activation mediated by LPS. In their revised manuscript, the authors addressed all my comments by adding additional experiments, clarifying the manuscript be it in the Results or Discussion sections and editing of unnecessary/overly complicated sentences. I want to stress that the authors responded very positively to my comments.
I appreciate the effort they made and I am happy to report that their manuscript is now appropriate for a publication in the Journal of Cell Science.

Comments for the author

I do not have any more comments.

Reviewer 2

Advance summary and potential significance to field

In this revised manuscript, the authors have addressed some major concerns of this reviewer. They included new findings showing the effect of BCAT1 inhibition is specific on glucose-derived TCA cycle metabolites which raises the possibility that the activity of TCA cycle enzymes is being targeted by BCAT1. The authors have also made efforts in linking the effect on TCA cycle to the redox control by BCAT1. Although there is no direct evidence yet to demonstrate such connection, the activation of Nrf2 pathway by BCAT1 inhibition indeed supports such possibility. Overall, while further investigation is required to elucidate clear mechanisms, the findings of the current manuscript do provide new insight into a possible moonlighting function of BCAT1.

Comments for the author

Specific points:

1. The authors mentioned in several places that BCAT1 inhibition induces ROS production (data not shown) that might indeed affect the TCA cycle activity. They should consider to include the findings as these could also explain why it activates Nrf2 in the first place.

The data in supplement figure 3 showing leucine is not metabolized into TCA cycle seem to be key findings that could support the alternative role of BCAT1 in controlling TCA cycle metabolism. If space is allowed, the authors should consider to include some of them, if not all, into the main figures.

In the discussion (page 12 paragraph 1 line 8), it states “among the TCA cycle enzymes influenced by BCAT1,...”. Does it refer to findings from previous studies? If so, the authors should include references here.

Second revision

Author response to reviewers' comments

- **Reviewer 1 Advance Summary and Potential Significance to Field:**

In the present work, Ko et al investigate the contribution of branched chain amino acid (BCAA) Leucine to the TCA cycle activation mediated by LPS. In their revised manuscript, the authors addressed all my comments by adding additional experiments, clarifying the manuscript be it in the Results or Discussion sections and editing of unnecessary/overly complicated sentences. I want to

© 2020. Published by The Company of Biologists under the terms of the Creative Commons Attribution License (https://creativecommons.org/licenses/by/4.0/).

stress that the authors responded very positively to my comments. I appreciate the effort they made and I am happy to report that their manuscript is now appropriate for a publication in the Journal of Cell Science.

Reviewer 1 Comments for the Author: I do not have any more comments.

We thank the reviewer whose comments and suggestions improved significantly the paper.

- **Reviewer 2 Advance Summary and Potential Significance to Field**

In this revised manuscript, the authors have addressed some major concerns of this reviewer. They included new findings showing the effect of BCAT1 inhibition is specific on glucose-derived TCA cycle metabolites, which raises the possibility that the activity of TCA cycle enzymes is being targeted by BCAT1. The authors have also made efforts in linking the effect on TCA cycle to the redox control by BCAT1. Although there is no direct evidence yet to demonstrate such connection, the activation of Nrf2 pathway by BCAT1 inhibition indeed supports such possibility. Overall, while further investigation is required to elucidate clear mechanisms, the findings of the current manuscript do provide new insight into a possible moonlighting function of BCAT1.

We thank the reviewer for the supportive and accurate review of the manuscript. All of the reviewers' suggestions were taken into consideration and the manuscript changed accordingly (see yellow highlighted text).

Reviewer 2 Comments for the Author:

Specific points:

The authors mentioned in several places that BCAT1 inhibition induces ROS production (data not shown) that might indeed affect the TCA cycle activity. They should consider to include the findings as these could also explain why it activates Nrf2 in the first place.

This is an excellent suggestion and we now include the data showing that a very short incubation with BCAT1 inhibitor ERG240 (30 min) causes a rise in mitochondrial ROS (Supplementary Figure 5d). We have therefore rectified the discussion sentences referring to this data as 'data not shown'. We have also included the quantification of mitoSOX in the methods.

The data in supplement figure 3 showing leucine is not metabolized into TCA cycle seem to be key findings that could support the alternative role of BCAT1 in controlling TCA cycle metabolism. If space is allowed, the authors should consider to include some of them, if not all, into the main figures.

We agree with the reviewer that the findings presented in supplementary Figure 3 are key to this manuscript. We follow the reviewer's advice and present them as part of a main figure (Figure 3a and b) in the revised manuscript. The order of the main and supplementary figures has been modified accordingly (highlighted text).

In the discussion (page 12 paragraph 1 line 8), it states "among the TCA cycle enzymes influenced by BCAT1,...". Does it refer to findings from previous studies? If so, the authors should include references here

We agree this sentence was not very clear and we have now modified into 'Indeed, among the TCA cycle enzymatic steps influenced by BCAT1, the citrate-isocitrate inter-conversion is catalysed by mitochondrial aconitase (ACO2), whose activity is tightly dependent on ROS levels'.

© 2020. Published by The Company of Biologists under the terms of the Creative Commons Attribution License (https://creativecommons.org/licenses/by/4.0/).

Third decision letter

MS ID#: JOCES/2020/247957

MS TITLE: BCAT1 affects mitochondrial metabolism independently of leucine transamination in activated human macrophages

AUTHORS: Jeong-Hun Ko, Antoni Olona, Adonia E. Papathanassiu, Norzawani Buang, Kwon-Sik Park, Ana S. H. Costa, Claudio Mauro, Christian Frezza, and Jacques Behmoaras
ARTICLE TYPE: Research Article

I am happy to tell you that your manuscript has been accepted for publication in Journal of Cell Science, pending standard ethics checks.

© 2020. Published by The Company of Biologists under the terms of the Creative Commons Attribution License (https://creativecommons.org/licenses/by/4.0/).
